# Tumour-associated macrophages secrete pleiotrophin to promote PTPRZ1 signalling in glioblastoma stem cells for tumour growth

**DOI:** 10.1038/ncomms15080

**Published:** 2017-06-01

**Authors:** Yu Shi, Yi-Fang Ping, Wenchao Zhou, Zhi-Cheng He, Cong Chen, Bai-Shi-Jiao Bian, Lin Zhang, Lu Chen, Xun Lan, Xian-Chao Zhang, Kai Zhou, Qing Liu, Hua Long, Ti-Wei Fu, Xiao-Ning Zhang, Mian-Fu Cao, Zhi Huang, Xiaoguang Fang, Xiuxing Wang, Hua Feng, Xiao-Hong Yao, Shi-Cang Yu, You-Hong Cui, Xia Zhang, Jeremy N Rich, Shideng Bao, Xiu-Wu Bian

**Affiliations:** 1 Institute of Pathology and Southwest Cancer Centre, Southwest Hospital, The Third Military Medical University, Chongqing 400038, China; 2 The Key Laboratory of Tumour Immunopathology, The Ministry of Education of China, Chongqing 400038, China; 3 Department of Stem Cell Biology and Regenerative Medicine, Lerner Research Institute, Cleveland Clinic, Cleveland, Ohio 44195, USA; 4 Department of Ophthalmology, Southwest Hospital, The Third Military Medical University, Chongqing 400038, China; 5 Department of Neurology, Xijing Hospital, The Fourth Military Medical University, Xi'an 710032, China; 6 Department of Genetics, Stanford University, Stanford, California 94305, USA; 7 Department of Neurosurgery, Southwest Hospital, The Third Military Medical University, Chongqing 400038, China; 8 Collaborative Innovation Center for Cancer Medicine, Sun Yat-sen University, Guangzhou 510095, China

## Abstract

Intense infiltration of tumour-associated macrophages (TAMs) facilitates malignant growth of glioblastoma (GBM), but the underlying mechanisms remain undefined. Herein, we report that TAMs secrete abundant pleiotrophin (PTN) to stimulate glioma stem cells (GSCs) through its receptor PTPRZ1 thus promoting GBM malignant growth through PTN–PTPRZ1 paracrine signalling. PTN expression correlates with infiltration of CD11b^+^/CD163^+^ TAMs and poor prognosis of GBM patients. Co-implantation of M2-like macrophages (MLCs) promoted GSC-driven tumour growth, but silencing PTN expression in MLCs mitigated their pro-tumorigenic activity. The PTN receptor PTPRZ1 is preferentially expressed in GSCs and also predicts GBM poor prognosis. Disrupting PTPRZ1 abrogated GSC maintenance and tumorigenic potential. Moreover, blocking the PTN–PTPRZ1 signalling by shRNA or anti-PTPRZ1 antibody potently suppressed GBM tumour growth and prolonged animal survival. Our study uncovered a critical molecular crosstalk between TAMs and GSCs through the PTN–PTPRZ1 paracrine signalling to support GBM malignant growth, indicating that targeting this signalling axis may have therapeutic potential.

## Abstract (web summary)

Tumour-associated macrophages (TAMs) facilitate malignant growth of glioblastoma (GBM). Here, the authors show that TAMs support glioma stem cell renewal via paracrine signalling to the pleiotrophin receptor PTPRZ1 and that blocking this axis results in increased survival of tumour-bearing animals.

Glioblastoma (GBM) is the most aggressive and lethal brain tumour that is highly resistant to conventional therapies^1^. It has been demonstrated that tumour microenvironment plays a critical role in supporting the malignant growth and progression of GBM^2^,^3^. The tumour microenvironment in GBM is composed of multiple components, including parenchyma cells, soluble factors, blood vessels, extracellular matrix and infiltrating immune cells^2^,^3^. As an important element of tumour microenvironment in GBMs, tumour-associated macrophages (TAMs) are enriched in GBMs and play crucial roles in supporting tumour growth^4^,^5^. TAM infiltration has been shown to correlate with glioma progression and tumour grade, and predicts poor survivals of GBM patients^6^,^7^. Recent studies suggested that TAMs can be functionally categorized into at least tumour-supportive (M2 type) macrophages and tumour suppressive (M1 type) macrophages^8^,^9^. While M1 TAMs display an immune surveillance function^9^,^10^, M2 TAMs are generally immune-suppressive and facilitate GBM malignant behaviours to promote tumour growth^11^,^12^. Targeting M2 TAMs potently attenuated GBM malignant progression in animals^13^,^14^, indicating that M2 TAMs are potential therapeutic targets for improving GBM treatment. Despite the crucial role of M2 TAMs in GBM malignancy, the molecular mechanisms underlying the pro-tumorigenic functions of M2 TAMs remain to be elucidated.

Recent studies indicated that TAMs actively communicate with tumour cells through producing soluble factors such as interleukin (IL)-6, IL-10 and transforming growth factor-β1 (refs 11, 15, 16). These paracrine cytokines are generally tumour-supportive, which activate tumour cell intrinsic signalling responsible for proliferation, invasion and vascularization. In addition, TAMs has been shown to be closely associated with glioma stem cells (GSCs), a subset of neoplastic cells that share stem cell-like properties and display potent tumour-initiating capacity to drive GBM malignant progression^17^,^18^. TAMs and GSCs are co-enriched in tumour perivascular niches, hypoxic regions and invasive fronts, suggesting a spatial functional link between TAMs and GSCs^11^,^19^,^20^. Moreover, both GSCs and TAMs have been reported to be increased in recurrent GBMs after irradiation^21^,^22^. The close association between TAMs and GSCs strongly suggests a reciprocal molecular crosstalk that is crucial for GBM malignant progression. Recently, we discovered that GSCs preferentially secreted periostin to recruit monocyte-derived TAMs from peripheral blood to GBM tumours^13^. However, how the GSC-recruited TAMs reciprocally facilitate GSC maintenance to promote GBM tumour propagation has not been defined.

To interrogate the mechanisms underlying the pro-tumorigenic functions of TAMs on GSCs, we screened for the soluble factors preferentially expressed by the CD11b^+^/CD163^+^ M2 TAMs isolated from human primary GBMs. We found that a heparin-binding glycoprotein pleiotrophin (PTN) was consistently and preferentially secreted by the CD11b^+^/CD163^+^ TAMs to promote GSC tumour growth. Consistently, the PTN receptor the protein tyrosine phosphatase, receptor-type, Z polypeptide 1 (PTPRZ1) was preferentially expressed by GSCs. PTN, also known as heparin-binding growth-associated molecule, is a critical cytokine that regulates diverse physiological functions^23^,^24^. Increased level of PTN has been detected in a number of malignant tumours^25^,^26^, and could predict poor prognosis of cancer patients^26^,^27^. PTN functions mainly through its receptor PTPRZ1 to increase phosphorylation of the downstream effectors, thereby activating the signal transduction related to cell growth, migration and cellular activities^28^,^29^,^30^,^31^. Similar to PTN, PTPRZ1 has also been found to be aberrantly expressed in various types of tumours^32^,^33^,^34^,^35^. However, the origin of PTN in GBMs and the role of PTN–PTPRZ1 signalling in regulating GSC properties remain unknown. In this study, we investigated the functional significance of the PTN–PTPRZ1 paracrine signalling in mediating the effects of TAMs on GSCs and tumour growth in GBMs, and identified the PTN–PTPRZ1 paracrine signalling axis as a critical molecular link between tumour-supportive TAMs and tumorigenic GSCs to facilitate GBM malignant progression.

## Results

### PTN is preferentially expressed by tumour-supportive TAMs

**Figure 1:**
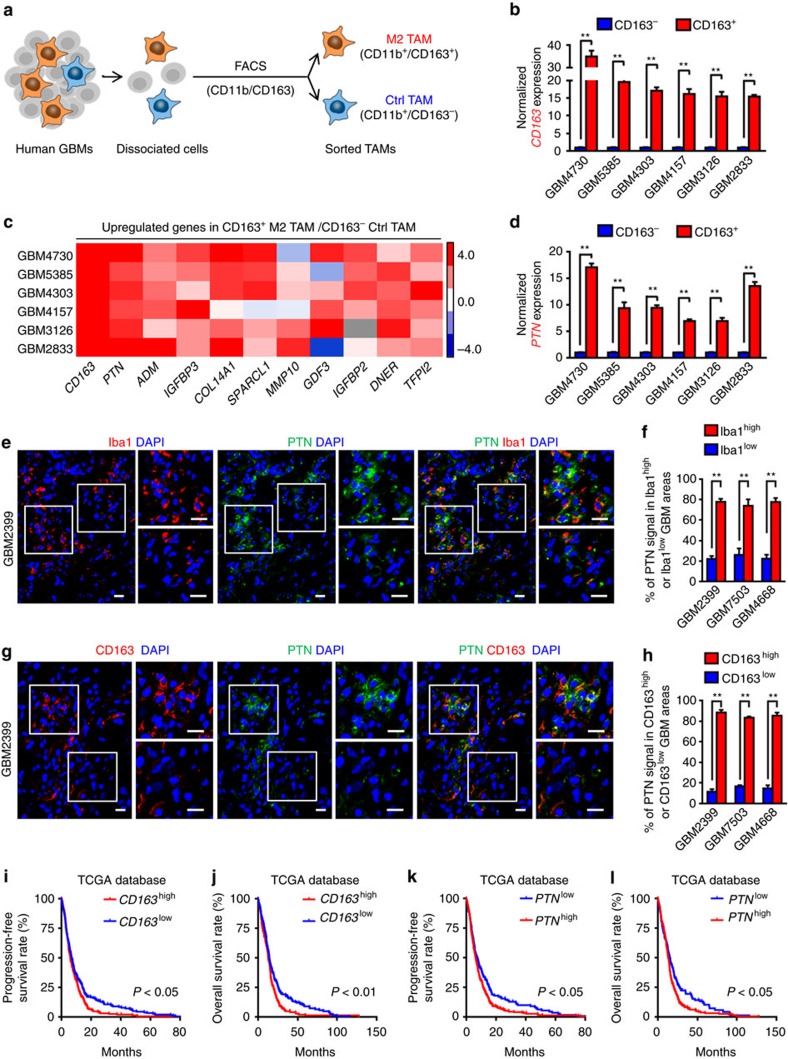
PTN is preferentially expressed in CD11b^+^/CD163^+^ M2 TAMs and informs poor prognosis of GBM patients. (**a**) Schematic diagram of the isolation of CD11b^+^/CD163^+^ M2 TAMs and CD11b^+^/CD163^−^ control TAMs from human GBMs using FACS. (**b**) qRT–PCR analyses of *CD163* expression between FACS-sorted CD11b^+^/CD163^+^ M2 TAMs and CD11b^+^/CD163^−^ control TAMs from six cases of human GBMs. Data are shown as means±s.e.m., *n*=3, **P<0.01, Student's *t*-test. (**c**) Expression heatmap of the upregulated genes in CD163^+^ M2 TAMs relative to CD163^−^ control TAMs sorted from human GBMs. The top 10 of gene candidates identified from the microarray profiling (GEO, GSE37475) were validated in CD11b^+^/CD163^+^ M2 TAMs relative to the CD11b^+^/CD163^−^ control TAMs using qRT–PCR analysis. *CD163* was used as a positive control marker. Data are shown as a heatmap using Cluster/Java Treeview. Heatmap colour ranging from minimum (blue) to maximum (red) represents the relative gene expression of CD163^+^ M2 TAMs to the CD163^−^ control TAMs.(**d**) qRT–PCR analyses of *PTN* expression between FACS-sorted CD11b^+^/CD163^+^ M2 TAMs and CD11b^+^/CD163^−^ control TAMs from six cases of human GBMs. Data are shown as means±s.e.m., *n*=3, **P<0.01, Student's *t*-test. (**e**) Representative immunofluorescent staining of the TAM marker Iba1 (in red) and PTN (in green) in human GBM tissues. Areas indicated with squares are enlarged and shown on the right side of each image. Scale bar represents 25 μm. (**f**) Graphical analysis of **e** showing abundant PTN expression in the Iba1^+^ TAM-enriched regions in GBMs. PTN expression and Iba1^+^ TAM density were quantified by ImageJ software. Data are shown as means±s.d., *n*=5, **P<0.01, Student's *t*-test. (**g**,**h**) Immunofluorescence (**g**) and corresponding quantification data (**h**) showing abundant PTN expression in the CD163^+^ TAM-enriched regions in human GBMs. Scale bar represents 25 μm. Data are shown as means±s.d., *n*=5, **P<0.01, Student's *t*-test. (**i**,**j**) Kaplan–Meier survival analysis of *CD163* expression and the progression-free survival (**i**) or overall survival (**j**) of GBM patients from the TCGA database. *P*<0.05 (**i**), *P*<0.01 (**j**), log-rank test. (**k**,**l**) Kaplan–Meier survival analysis of *PTN* expression and the progression-free survival (**k**) or overall survival (**l**) of GBM patients from the TCGA database. *P*<0.05, log-rank test. DAPI, 4,6-diamidino-2-phenylindole.

To screen for the M2 TAM-secreted factors potentially associated with GBM malignant growth, we analysed the microarray data of the TAMs with M2 suppression (M2^low^ TAMs) and the TAMs without M2 suppression (M2^high^ TAMs) isolated from murine gliomas (Gene Expression Omnibus (GEO) database, GSE37475)^14^. A total of 258 molecules were identified to be differentially expressed (fold change ≥2.0) between these TAM populations (Supplementary Data 1). We overlapped the differentially expressed molecules between M2^high^ and M2^low^ populations with mouse-secreted proteins from the Secreted Protein Database^36^, and identified 20 soluble factors whose expressions were reduced in M2^low^ TAMs relative to M2^high^ TAMs (Supplementary Fig. 1a,b). Next, we examined the expressions of these candidate factors in infiltrating TAMs in human GBMs. We used a well-known pan TAM marker CD11b and a classical M2 TAM marker CD163 in fluorescence-activated cell sorting (FACS) to obtain the tumour-supportive M2 TAMs (CD11b^+^/CD163^+^) and the control TAMs (CD11b^+^/CD163^−^) (Fig. 1a). Increased expression of CD163 in FACS-sorted CD163^+^ TAMs relative to the CD163^−^ TAMs was confirmed by quantitative real-time–PCR (Fig. 1b, *P*<0.01, Student's *t*-test). Meanwhile, among the top 10 of the M2 TAM-associated factors identified from the microarray analysis, the heparin-binding factor PTN, but not adrenomedullin (ADM), COL14A1 or IGFBP3, was most consistently upregulated (fold change >6.0) in M2 TAMs relative to the control TAMs in all tested GBM specimens (Fig. 1c,d and Supplementary Fig. 1c–e). To determine PTN distribution and further confirm its correlation with TAMs in GBMs, frozen tumour sections were co-immunostained with PTN and the pan TAM marker Iba1 or the M2 TAM marker CD163. We found that the tumour areas with more Iba1^+^ TAM infiltration showed more PTN staining (Fig. 1e,f and Supplementary Fig. 1f, *P*<0.01, Student's *t*-test). Furthermore, the distribution of PTN clearly correlated with the infiltration of the CD163^+^ M2 TAMs (Fig. 1g,h and Supplementary Fig. 1g, *P*<0.01, Student's *t*-test). Consistently, immunohistochemical staining (IHC) and bioinformatic analyses demonstrated a positive correlation between PTN and CD163 in GBMs (Supplementary Fig. 1h,i and Supplementary Table 1). These data demonstrate that PTN is preferentially expressed by the CD163^+^ M2 TAMs in human GBMs.

To interrogate the clinical significance of TAM infiltration and PTN expression in GBMs, we used the Cancer Genome Atlas (TCGA) database to analyse the potential associations between GBM patient survival and the expression of pan TAM marker Iba1, the M2 TAM marker CD163 or PTN. The Kaplan–Meier analyses demonstrated that expression of Iba1 or CD163 negatively correlated to GBM patient survival (Fig. 1i,j and Supplementary Fig. 1j,k), suggesting that higher TAM infiltration contributes to more aggressive GBMs. Significantly, higher PTN expression informed poorer progression-free survival and overall survival in GBM patients (Fig. 1k,l), suggesting a potential pro-tumorigenic effect of PTN in promoting GBM malignancy. Collectively, these data demonstrate that PTN is preferentially secreted by M2 TAMs and its expression is correlated with poor survival of GBM patients.

### PTN mediates the pro-tumorigenic effect of M2 TAMs

**Figure 2:**
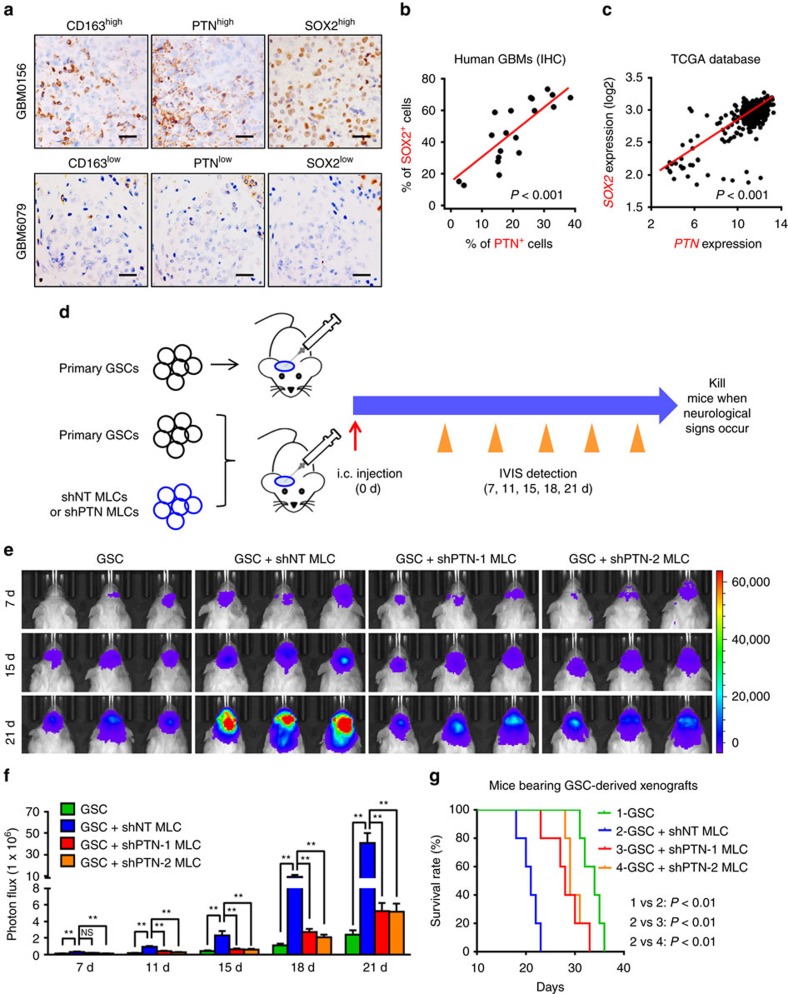
PTN is enriched with GSCs and mediates the pro-tumorigenic effect of M2 TAMs. (**a**) IHC staining of M2 TAM marker CD163, PTN and the GSC marker SOX2 in human GBMs using serial sections. Scale bar represents 50 μm. (**b**,**c**) Correlation analysis of PTN and SOX2 expressions in GBM specimens from the Southwest hospital (**b**) and those from the TCGA database (**c**). *P*<0.001, Pearson's *r* test. (**d**) Schematic diagram of GSC-driven xenografts co-implanted with U937-derived MLCs expressing shPTN (shPTN MLCs) or shNT (shNT MLCs). After implantation, tumour growth was monitored through the IVIS bioluminescent imaging system and mice were killed when neurological signs occurred. (**e**–**f**) The representative bioluminescent images (**e**) and the quantification (**f**) of the tumour-bearing mice implanted with GSCs only or co-implanted with GSCs and shPTN MLCs or shNT MLCs. Data are shown as means ±s.e.m., *n*=5, **P<0.01, NS, not significant, ANOVA test. (**g**) Kaplan–Meier survival curves of mice implanted with GSCs only or co-implanted with GSCs and shPTN MLCs or shNT MLCs. *n*=5, *P*<0.01, log-rank test. d, days.

TAMs have been shown to be recruited by GSCs and are maintained as M2 TAMs to promote GBM tumour growth^13^,^14^. To address whether M2 TAMs exert their tumour-promoting effect through PTN, we examined the potential co-distribution between PTN and GSCs in serial sections of human GBM specimens. IHC staining of PTN, the GSC marker SOX2 and the M2 TAM marker CD163 revealed that tumour regions with high levels of PTN expression and CD163^+^ M2 TAM infiltration contained abundant GSCs marked by SOX2 (Fig. 2a). In contrast, PTN expression and the infiltration of CD163^+^ M2 TAMs were markedly reduced in tumour areas lacking GSCs (Fig. 2a), suggesting a co-distribution among GSCs, M2 TAMs and PTN. The positive correlation between PTN and SOX2 was further confirmed in our GBM specimens (*n*=20; *P*<0.001, Pearson's *r* test; Fig. 2b and Supplementary Table 1) and those from the TCGA database (*n*=541; *P*<0.001, Pearson's *r* test; Fig. 2c). Thus, the M2 TAM-expressed PTN is co-enriched with GSCs in human GBMs, indicating a potential role of PTN in maintaining the stem cell-like properties and tumorigenic potential of GSCs.

To address whether the M2 TAM-secreted PTN functions as a paracrine factor to promote GSC tumour growth, we investigated whether silencing PTN expression in the M2-like TAMs could attenuate the pro-tumorigenic effect of TAMs on GSC-driven tumour growth. Since primed U937 cells possess major macrophage characteristics and have been widely used as a macrophage model for many studies^13^,^37^,^38^, we polarized U937 cells into M2 MLCs to mimic TAMs for our *in vivo* study^39^ (Supplementary Fig. 2a). The M2-like phenotype of the MLCs was confirmed by the markedly elevated expressions of the M2 markers and the reduced expressions of the M1 markers (Supplementary Fig. 2b,c). Of note, the induction of the M2-like phenotype in U937 cells also significantly elevated PTN expression (Supplementary Fig. 2b–e, *P*<0.01, Student's *t*-test). Importantly, co-implantation of the MLCs with GSCs potently promoted GSC tumour growth *in vivo*, suggesting that the primed MLCs mimicked the pro-tumorigenic properties of M2 TAMs (Fig. 2d–f and Supplementary Fig. 2j, *P*<0.01, analysis of variance (ANOVA) test). Next we investigated whether disrupting PTN expression could abolish the tumour-promoting effect of MLCs in GSC xenografts. Introduction of short hairpin RNAs (shRNAs) against PTN (shPTNs) in MLCs reduced PTN expression to 5.5–12.7% relative to the non-targeting control shRNA (shNT; Supplementary Fig. 2f, *P*<0.01, ANOVA test), which was confirmed by immunofluorescent analyses (Supplementary Fig. 2g,h). Disruption of PTN expression did not affect the viability of MLCs (Supplementary Fig. 2i), suggesting that PTN may not be required for MLC maintenance. We then co-implanted MLCs expressing shPTN (shPTN MLCs) or control shRNA (shNT MLCs) with the GSCs expressing luciferase into mouse brains (Fig. 2d). The immunofluorescent analyses confirmed the presence of U937-derived MLCs labelled by mCherry as a tracing marker in GBM xenografts (Supplementary Fig. 3a,b). Significantly, bioluminescent imaging and haematoxylin and eosin staining demonstrated that silencing PTN expression in MLCs significantly compromised the tumour-promoting effect of the co-implanted MLCs, resulting in the retarded tumour growth in GBM xenografts co-implanted with GSCs and shPTN MLCs relative to those co-implanted with GSCs and shNT MLCs (Fig. 2e,f and Supplementary Figs 2j and 3c). The IHC analyses of PTN confirmed that PTN expression was significantly increased in the xenografts co-implanted with GSCs and shNT MLCs, but was reduced in the xenografts derived from GSCs and shPTN MLCs (Supplementary Fig. 3d,e, *P*<0.01, ANOVA test). These results confirmed that TAM-secreted PTN was indispensable for the pro-tumorigenic function of TAMs. To investigate whether implanted MLCs could affect GSC maintenance, we evaluated the expression of GSC marker SOX2 in the xenografts. The percentage of GSCs marked by SOX2 in xenografts derived from GSCs and shNT MLCs was higher than that in the control xenografts derived from GSCs (Supplementary Fig. 3f,g, *P*<0.01, ANOVA test). Meanwhile, disrupting PTN in MLCs reduced the proportion of SOX2^+^ tumour cells in the xenografts co-implanted with GSCs and shPTN MLCs (Supplementary Fig. 3f,g, *P*<0.01, ANOVA test), suggesting that TAM-secreted PTN promoted GSC maintenance. Consequently, mice co-implanted with GSCs and shNT MLCs had significantly shortened survival relative to those implanted with GSCs only (Fig. 2g, *P*<0.01, log-rank test). However, disruption of PTN in MLCs markedly attenuated such effect of shNT MLCs on animal survival (Fig. 2g, *P*<0.01, log-rank test). Taken together, these data demonstrate that the M2 TAM-secreted PTN is a crucial paracrine factor in mediating the tumour-supportive effect of TAMs to promote GSC tumour growth.

### The PTN receptor PTPRZ1 is preferentially expressed by GSCs

**Figure 3:**
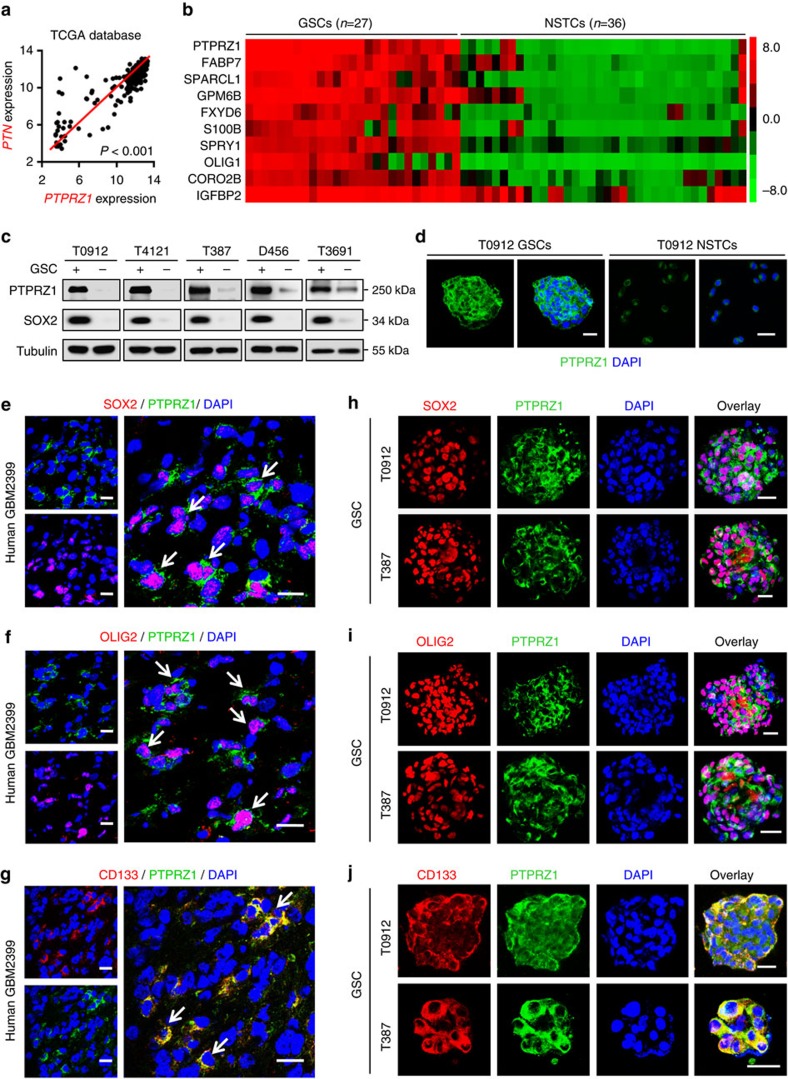
The PTN receptor PTPRZ1 is preferentially expressed by GSCs. (**a**) Bivariate correlation analyses showing a positive correlation of *PTN* and *PTPRZ1* expression in human GBMs from the TCGA database (https://tcga-data.nci.nih.gov/tcga). The expressions of *PTN* and *PTPRZ1* in human GBMs were obtained from gene-profiling data from the TCGA database. *n*=541, *P*<0.001, Pearson's *r* test. (**b**) Expression heatmap of top 10 upregulated genes in GSC lines (*n*=27) relative to NSTC lines (*n*=36) from GEO profiles (GEO: GDS3885). Candidate genes enriched in GSCs were identified when the average gene expression ratio of GSCs/NSTCs was ≥3.0-fold. Heatmap was visualized using Cluster/Java Treeview. (**c**) Immunoblot analysis showing preferential expression of PTPRZ1 and the GSC marker SOX2 in GSCs relative to matched NSTCs derived from human GBMs. α-Tubulin was used for normalization. (**d**) Immunofluorescence of PTPRZ1 (in green) in GSCs or NSTCs from T0912 GBM. Scale bar represents 50 μm. (**e**–**g**) Co-immunofluorescent staining of PTPRZ1 (in green) and the GSC marker SOX2 (in red, **e**), OLIG2 (in red, **f**) and CD133 (in red, **g**) in human GBMs. PTPRZ1 is preferentially expressed in GBM cells expressing GSC markers. Scale bar represents 25 μm. (**h**–**j**) Co-immunofluorescent staining of PTPRZ1 (in green) and the GSC marker SOX2 (in red, **h**), OLIG2 (in red, **i**) or CD133 (in red, **j**) in GSC tumourspheres. Co-enrichment of PTPRZ1 with the GSC markers was observed in GSC tumourspheres. Scale bar represents 25 μm. DAPI, 4,6-diamidino-2-phenylindole.

To determine the signalling pathway that mediates the tumour-supportive effect of PTN on GSCs, we examined the expression of the PTN receptor on GSCs. PTPRZ1 has been identified as a critical receptor of PTN^28^,^29^. Immunofluorescent stainings of PTN and PTPRZ1, and *in silico* analysis confirmed a positive correlation between PTPRZ1 and PTN in GBMs (Supplementary Fig. 4a,b and Fig. 3a, *P*<0.001, Pearson's *r* test), indicating a potential role of PTPRZ1 in mediating the PTN functions. To determine the expression of PTPRZ1 in GSCs, we used two independent GEO microarray data sets (GDS3885 and GSE54791) and found that *PTPRZ1* was one of the 83 genes significantly upregulated in GSCs (average fold changes >3.0) relative to the matched non-tumour stem cells (NSTCs) (Fig. 3b and Supplementary Table 2). Consistently, elevated expression of PTPRZ1 in GSCs was validated by immunoblot, immunofluorescence and flow cytometry analyses in established GSCs and matched NSTCs isolated from human GBMs (Fig. 3c,d and Supplementary Fig. 4c,d)^13^,^40^,^41^,^42^,^43^. To investigate PTPRZ1 expression pattern in GBMs, we co-immunostained PTRPZ1 and several GSC markers in frozen GBM specimens and found a preferential expression of PTPRZ1 in tumour cells expressing GSC markers SOX2, OLIG2 and CD133 (Fig. 3e–g and Supplementary Fig. 4e). Co-expression of PTPRZ1 with SOX2, OLIG2 or CD133 was also detected in GSC tumourspheres (Fig. 3h–j). Consistently, *in silico* analyses confirmed that mRNA level of *PTPRZ1* positively correlated with the GSC marker *SOX2* in GBMs (Supplementary Fig. 4f, *P*<0.001, Pearson's *r* test). In addition, the Kaplan–Meier survival analysis of GBM patients from the TCGA database demonstrated an inverse correlation between PTPRZ1 expression and GBM patient survival (Supplementary Fig. 4g,h). The combined PTPRZ1 and PTN expression is more significant to predict the outcomes of GBM patients (Supplementary Fig. 4i,j), suggesting that PTPRZ1 and PTN may serve as a prognostic biomarker set for GBM patients. Taken together, these data demonstrate that PTPRZ1 is preferentially expressed in GSCs and may mediate the effects of PTN on GSC maintenance.

### The PTN–PTPRZ1 signalling is crucial for GSC maintenance

**Figure 4:**
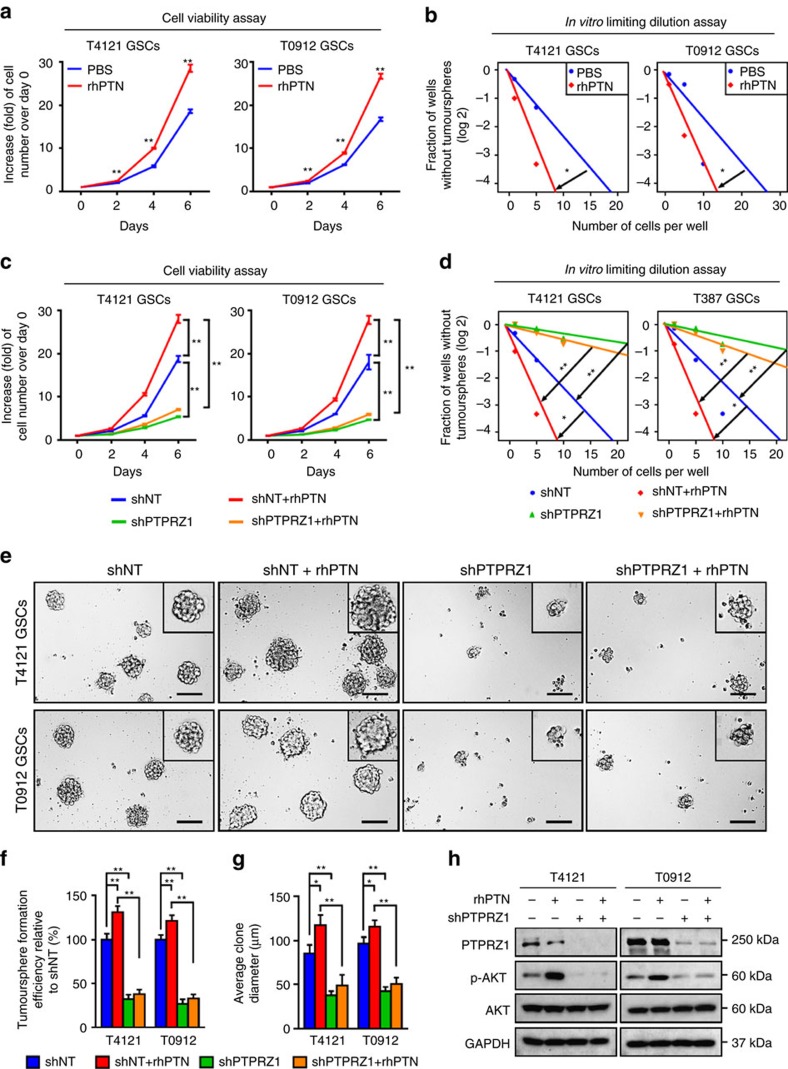
The PTN–PTPRZ1 signalling axis is critical for GSC maintenance. (**a**,**b**) The cell viability assay (**a**) and *in vitro* limiting dilution assay (**b**) of GSCs treated with rhPTN showing that rhPTN facilitated GSC proliferation and self-renewal. Data are shown as means±s.d., *n*=5, **P<0.01, Student's *t*-test (**a**); *n*=10, *P<0.05, likelihood ratio test (**b**). (**c**,**d**) The cell viability assay (**c**) and *in vitro* limiting dilution assay (**d**) of GSCs expressing shPTPRZ1 or shNT in combination of rhPTN treatment. Disruption of PTPRZ1 compromised the promoting role of PTN on GSC proliferation (**c**) and self-renewal (**d**). Data are shown as means±s.d., *n*=5, **P<0.01, Student's *t*-test (**c**); *n*=10, *P<0.05, **P<0.01, likelihood ratio test (**d**). (**e**–**g**) Tumoursphere formation assay of GSCs expressing shPTPRZ1 or shNT in combination with the treatment of rhPTN. The representative images of GSC tumourspheres (**e**) and the quantification of numbers (**f**) and diameter (**g**) of the GSC tumourspheres showing that silencing PTPRZ1 expression significantly compromises rhPTN-promoted GSC tumoursphere formation ability. Scale bar represents 100 μm. Data are shown as means±s.d., *n*=5, *P<0.05, **P<0.01, ANOVA test. (**h**) Immunoblot analyses of PTPRZ1, phospho-AKT (p-Ser473) and total AKT in GSCs expressing shPTPRZ1 or shNT in combination with rhPTN stimulation.

Since PTPRZ1 is preferentially expressed in GSCs, we investigated the potential role of the PTN–PTPRZ1 signalling in GSC maintenance. Cell viability assays and *in vitro* limiting dilution assays showed that recombinant human PTN (rhPTN) promoted GSC proliferation and self-renewal (Fig. 4a,b). However, disruption of the PTN–PTPRZ1 signalling by shRNAs against PTPRZ1 in GSCs (Supplementary Fig. 5a–d) compromised the promoting role of PTN (Fig. 4c,d), indicating that PTPRZ1 is crucial for the effect of PTN on the GSC maintenance. The critical role of PTPRZ1 was further confirmed by tumoursphere formation assays as disruption of PTPRZ1 potently inhibited the promoting effect of PTN on GSC tumoursphere formation (Fig. 4e–g). To confirm the significance of PTN–PTPRZ1 signalling to mediate the supportive role of TAMs on GSC growth, we used the conditioned medium of MLCs and determined its effect on GSC maintenance. The cell viability analyses demonstrated that the proliferation of GSCs cultured in the MLC conditioned medium was faster than those cultured in the control medium (Supplementary Fig. 5e, *P*<0.01, ANOVA test). However, the promoting effect of MLC conditioned medium on GSC growth was largely impaired by the PTPRZ1 disruption in GSCs (Supplementary Fig. 5e, *P*<0.01, ANOVA test). Mechanically, as AKT pathway has been reported to be regulated by PTN–PTPRZ1 signalling^44^, we investigated the role of PTPRZ1 in AKT activation on PTN stimulation. As expected, rhPTN treatment markedly induced AKT phosphorylation (p-Ser473) in GSCs, whereas disruption of PTPRZ1 largely abrogated the rhPTN-induced AKT activation (Fig. 4h). Collectively, these data demonstrate that PTN–PTPRZ1 signalling is crucial for mediating the supportive effect of TAMs on GSC maintenance.

### Disrupting PTPRZ1 potently inhibits GSC tumour growth

**Figure 5:**
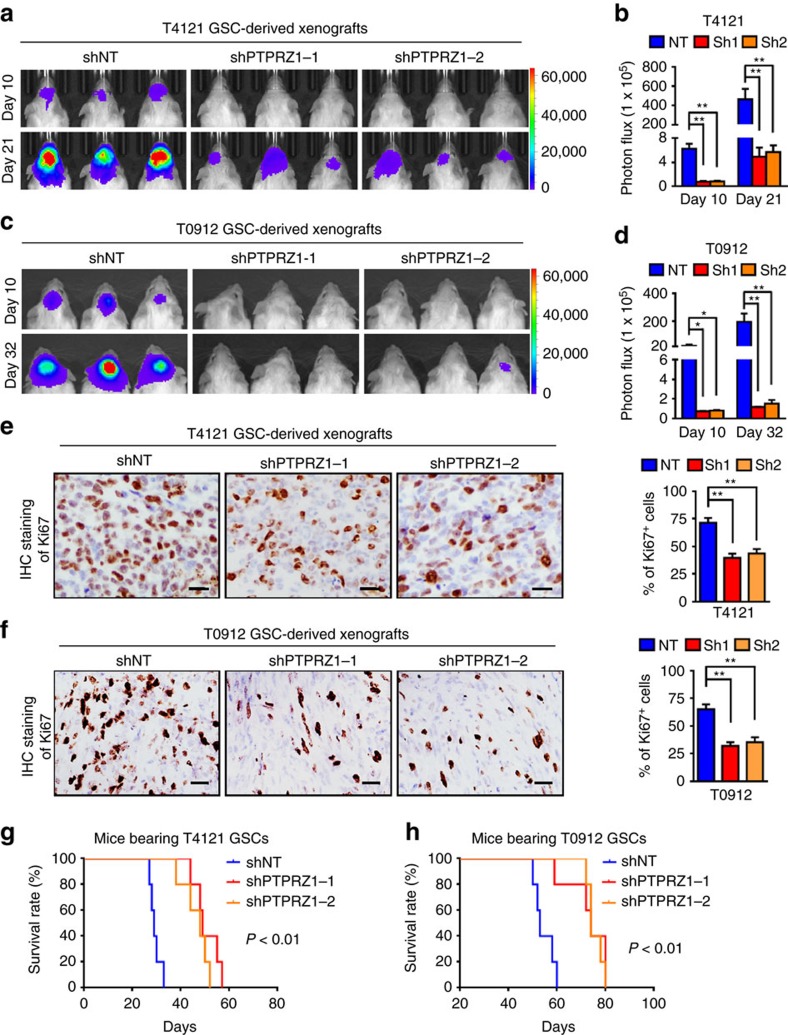
Disruption of PTPRZ1 potently inhibits GSC tumour growth and prolongs the animal survival. (**a**–**d**) The bioluminescent images (**a**,**c**) and quantification (**b**,**d**) of xenografts derived from T4121 GSCs (**a**,**b**) or T0912 GSCs (**c**,**d**) expressing shPTPRZ1 or shNT control. Data are shown as means±s.e.m., *n*=5, *P<0.05, **P<0.01, ANOVA test. (**e**,**f**) The representative IHC images (left panel) and the quantification (right panel) of Ki67 expression in xenografts derived from T4121 GSCs (**e**) or T0912 GSCs (**f**) expressing shPTPRZ1 or shNT control. Xenografts were collected at 28 days (T4121) or 56 days (T0912) after tumour implantation. Data are shown as means±s.d., *n*=5, **P<0.01, ANOVA test. Scale bar represents 25 μm. (**g**,**h**) Kaplan–Meier survival curves of mice implanted with T4121 GSCs (**g**) or T0912 GSCs (**h**) expressing shPTPRZ1 or shNT control. Disruption of PTPRZ1 significantly extends the survival of mice bearing GSC-derived xenografts. *n*=5, *P*<0.01, log-rank test.

To interrogate the impact of the PTN–PTPRZ1 signalling on GSC-driven tumour propagation, we examined the effect of PTPRZ1 disruption on GSC tumour growth. GSCs (T4121 and T0912) expressing luciferase and shPTPRZ1 or control shNT were implanted into mouse brains. IHC analyses confirmed a significant reduction of PTPRZ1 in the xenografts expressing shPTPRZ1 relative to the control xenografts (Supplementary Fig. 6a,b, *P*<0.01, ANOVA test). Importantly, bioluminescent analyses showed that PTPRZ1 disruption potently inhibited GSC tumour growth (Fig. 5a–d). IHC staining of Ki67 confirmed the reduced cell proliferation in the shPTPRZ1-expressing xenografts relative to the control tumours (Fig. 5e,f, *P*<0.01, ANOVA test). To investigate whether disrupting PTPRZ1 expression in GSCs affected TAM infiltration, we performed immunofluorescent analyses of M2 TAM marker CD163 in GBM xenografts. The percentage of M2 TAMs marked by CD163 was reduced in the shPTPRZ1 xenografts (Supplementary Fig. 6c,d, *P*<0.05, ANOVA test), suggesting that disrupting PTPRZ1 impaired TAM infiltration. We also found that the fraction of GSCs marked by SOX2 was reduced in the xenografts expressing shPTPRZ1 (Supplementary Fig. 6e,f, *P*<0.01, ANOVA test), indicating that silencing PTPRZ1 expression disrupted GSC maintenance. The association between the reduced GSC population and the impaired TAM infiltration in shPTPRZ1 xenografts supported our previous findings, showing that GSCs are privileged to recruit peripheral TAMs in to GBMs thus promote tumour growth^13^. Moreover, mice bearing shPTPRZ1-expressing xenografts exhibited extended survivals relative to those bearing the control tumours (Fig. 5g,h, *P*<0.01, log-rank test). Collectively, these data demonstrate that disruption of PTPRZ1 effectively attenuates the tumour-propagating capacity of GSCs.

To address whether the pro-tumorigenic effect of TAMs is exerted through PTPRZ1 expressed on GSCs, we determined the effect of PTN-expressing MLCs on the growth of PTPRZ1^+^ and PTPRZ1^−^ tumours. The bioluminescent imaging analyses demonstrated that co-implanted MLCs significantly promoted the growth of PTPRZ1^+^ tumours (Supplementary Fig. 7a,b), while the supportive effect of MLCs on the PTPRZ1^−^ tumours was less significant (Supplementary Fig. 7a,b). These results indicate that PTPRZ1 expression in glioma cells is required to mediate the tumour-supportive role of TAMs. Consequently, the survival of mice co-implanted with PTPRZ1^+^ glioma cells and MLCs was shorter than that of the mice implanted with PTPRZ1^+^ glioma cells alone (Supplementary Fig. 7c). Meanwhile, no significant difference on mouse survival was found between the mice implanted with PTPRZ1^−^ glioma cells alone and those co-implanted with PTPRZ1^−^ glioma cells and MLCs (Supplementary Fig. 7c). These results confirmed that TAM-secreted PTN exerts its tumour-promoting function through PTPRZ1 expressed on GSCs.

### Glioma cells with high levels of PTPRZ1 are enriched with GSCs

**Figure 6:**
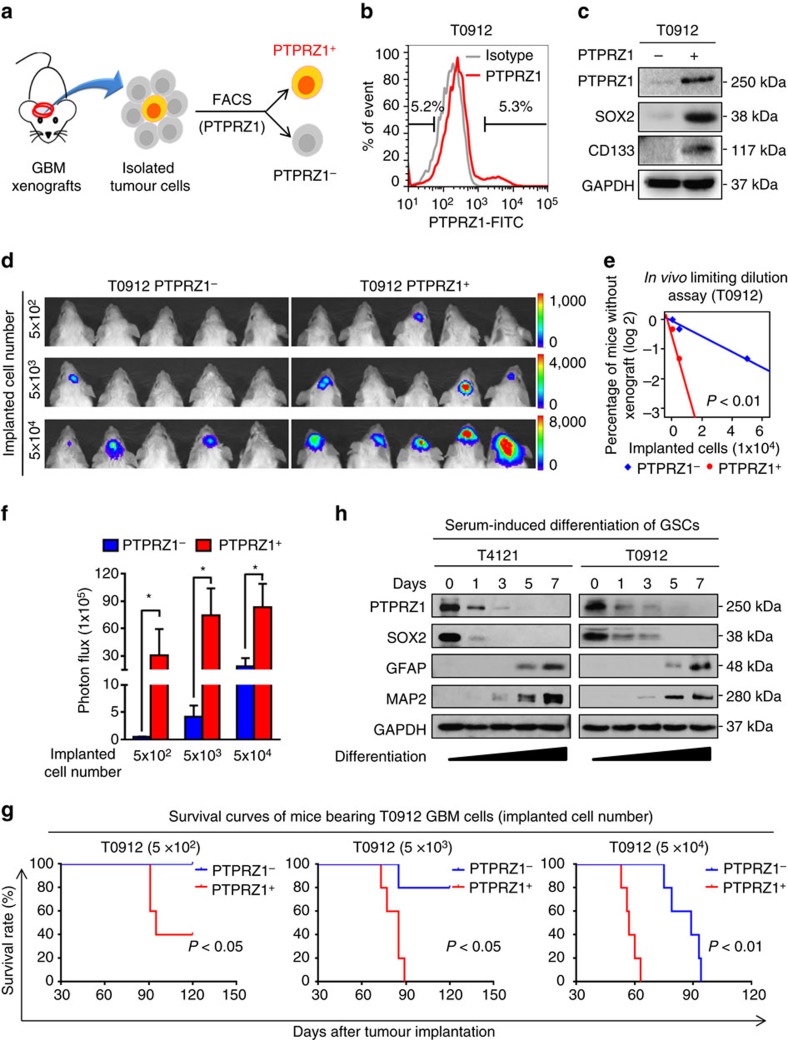
GSCs are enriched in the glioma cells with high PTPRZ1 expression. (**a**) Schematic diagram of the isolation of PTPRZ1^+^ and PTPRZ1^−^ subpopulations from orthotopic GBM xenografts. (**b**) FACS sorting of PTPRZ1^+^ and PTPRZ1^−^ subpopulations from T0912 GBM xenografts. Human GBM cells dissociated from xenografts were enriched and incubated with anti-human PTPRZ1 antibody or the isotype IgG followed by incubation of FITC-labelled secondary antibody to sort PTPRZ1^+^ and PTPRZ1^−^ GBM cells. (**c**) Immunoblot analyses of the expressions of PTPRZ1 and GSC markers (SOX2 and CD133) in FACS-sorted PTPRZ1^+^ and PTPRZ1^−^ glioma cells. (**d**) Representative bioluminescent images of intracranial GBM xenografts derived from FACS-sorted PTPRZ1^+^ and PTPRZ1^−^ glioma cells. Tumour status was detected by IVIS bioluminescent imaging. *n*=5. (**e**) *In vivo* limiting dilution assay showing that PTPRZ1^+^ glioma cells exhibit a higher tumour formation incidence than matched PTPRZ1^−^ glioma cells. The tumour formation incidence was determined through IVIS bioluminescent imaging, and the tumour formation efficiency was calculated using Extreme limiting dilution analysis (http://bioinf.wehi.edu.au/software/elda/). *n*=5, *P*<0.01, likelihood ratio test. (**f**) Bioluminescent quantification of intracranial GBM xenografts in **d**. Data are shown as means±s.e.m., *n*=5, *P<0.05, Student's *t*-test. (**g**) Kaplan–Meier survival analysis of mice implanted with indicated numbers of PTPRZ1^+^ or PTPRZ1^−^ glioma cells isolated from T0912 GBM. Mice implanted with PTPRZ1^+^ glioma cells exhibit reduced survival. *n*=5, log-rank test. (**h**) Immunoblot analyses of PTPRZ1, the GSC marker SOX2, the astrocytic differentiation marker GFAP and the neuronal differentiation marker MAP2 in GSCs cultured in serum-induced differentiation medium over a 7-day period. Expressions of PTPRZ1 and the GSC marker SOX2 decrease, while expressions of the differentiation markers GFAP and MAP2 concomitantly increase.

Given the critical role of PTPRZ1 in maintaining the GSC phenotype, high expression of PTPRZ1 may predict an enrichment of GSC population in GBM. To test this possibility, we isolated the PTPRZ1^+^ and PTPRZ1^−^ subpopulations from primary T0912 GBM xenografts (Fig. 6a,b). Preferential expressions of the GSC marker SOX2 and CD133 were detected in the PTPRZ1^+^ subpopulation relative to the PTPRZ1^−^ subpopulation (Fig. 6c), indicating an enrichment of GSCs in the PTPRZ1^+^ subpopulation. Consistently, *in vivo* limiting dilution assays showed that as low as 500 PTPRZ1^+^ glioma cells were sufficient to initiate the intracranial tumour growth, whereas a minimum of 5 × 10^3^ PTPRZ1^−^ glioma cells were required for tumour formation (Fig. 6d). Statistical analyses of the tumour formation incidence showed that PTPRZ1^+^ cells initiated tumours with a higher frequency relative to the matched PTPRZ1^−^ population (Fig. 6e). Moreover, mice bearing PTPRZ1^+^ cell-derived tumours displayed much more rapid tumour growth than mice implanted with PTPRZ1^−^ cells (Fig. 6f, *P*<0.05, Student's *t*-test). Consequently, mice bearing the PTPRZ1^+^ xenografts exhibited markedly shortened survivals relative to those bearing the PTPRZ1^−^ tumours (Fig. 6g). These results demonstrate that the PTPRZ1^+^ subpopulation is enriched with GSCs and displays more potent tumorigenic capacity than the PTPRZ1^−^ subpopulation. Since GSC population diminishes during the serum-induced differentiation, we investigated the change of PTPRZ1 expression during GSC differentiation. The decrease of GSC population was demonstrated by the gradual loss of GSC marker SOX2 along with the increase of astrocytic marker glial fibrillary acidic protein (GFAP) and neuronal marker microtubule-associated protein 2 (MAP2) (Fig. 6h). Meanwhile, a gradual decrease of PTPRZ1 was observed during the serum-induced GSC differentiation, indicating a close association between PTPRZ1 expression and the GSC status (Fig. 6h and Supplementary Fig. 7d). Taken together, these data suggest that high PTPRZ1 expression predicts enrichment of GSCs with stem cell-like properties and potent tumorigenic capability.

### PTN-PTPRZ1 axis is essential for GSC tumour propagation

**Figure 7:**
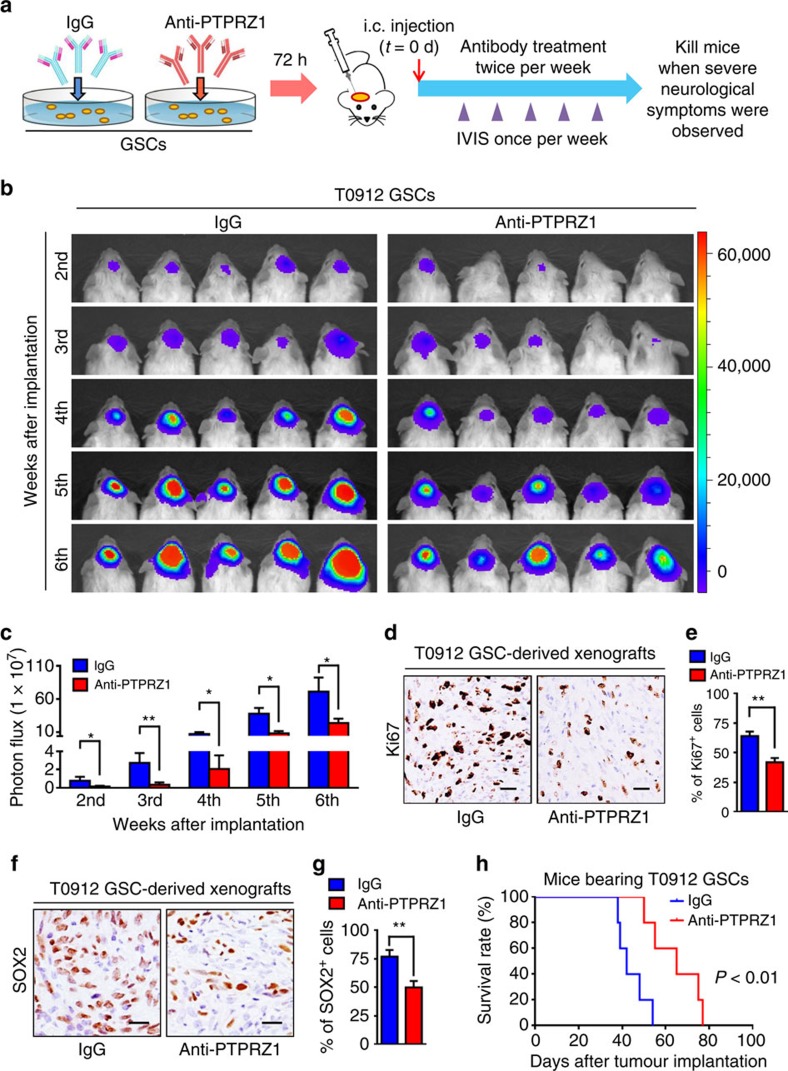
Treatment with the anti-PTPRZ1 antibody potently inhibits GSC tumour growth and extends animal survival. (**a**) Schematic diagram showing the treatment of GSCs and the GSC-derived xenografts with the anti-PTPRZ1 antibody. GSCs were incubated with the anti-PTPRZ1 antibody (5 μg ml^−1^) or the isotype control IgG for 72 h followed by orthotopic implantation. After GSC transplantation, mice were treated with the anti-PTPRZ1 antibody (intravenous (i.v.), 2 mg kg^−1^) or isotype IgG twice per week until moribund. (**b**,**c**) Representative bioluminescent images (**b**) and the quantification (**c**) of intracranial xenografts derived from T0912 GSCs treated with anti-PTPRZ1 antibody or IgG control at the indicated weeks after GSC transplantation. Data are shown as means±s.e.m., *n*=5, *P<0.05, **P<0.01, Student's *t*-test. (**d**,**e**) Representative IHC images (**d**) and the quantification (**e**) of Ki67 in the xenografts treated with anti-PTPRZ1 antibody or IgG control. The anti-PTPRZ1 antibody treatment significantly inhibited GBM proliferation in the GSC-derived xenografts. Scale bar represents 25 μm. Data are shown as means±s.d., *n*=5, **P<0.01, Student's *t*-test. (**f**,**g**) Representative IHC images (**f**) and quantification (**g**) of SOX2-positive cells in the T0912 xenografts treated with anti-PTPRZ1 antibody or IgG control. Scale bar represents 25 μm. Data are shown as means±s.d., *n*=5, **P<0.01, Student's *t*-test. (**h**) Kaplan–Meier survival curves of the mice bearing the GSC-derived xenografts treated with anti-PTPRZ1 antibody or IgG control. The anti-PTPRZ1 antibody treatment significantly prolonged the survival of mice bearing the GBM xenografts. *n*=5, *P*<0.01, log-rank test. d, day.

To evaluate the therapeutic potential of targeting the PTN–PTPRZ1 paracrine signalling in GBM treatment, we examined whether a specific anti-PTPRZ1 antibody could inhibit GSC-derived tumour growth. GSCs (T0912) expressing luciferase were incubated with the anti-PTPRZ1 antibody or control IgG and then implanted into mouse brains followed by intravenous administration of anti-PTPRZ1 antibody or the isotype IgG throughout the period of tumour growth (Fig. 7a). Bioluminescence analyses demonstrated that the anti-PTPRZ1 antibody treatment markedly retarded GSC tumour growth (Fig. 7b,c). Consistently, a significant reduction of Ki67-positive proliferating cells was detected in the anti-PTPRZ1 antibody-treated xenografts (Fig. 7d,e, *P*<0.01, Student's *t*-test). We also found that the GSC population marked by SOX2 staining was decreased in xenografts treated with anti-PTPRZ1 antibody relative to those treated with IgG control (Fig. 7f,g, *P*<0.01, Student's *t*-test), suggesting that targeting PTPRZ1 signalling disrupted GSC maintenance. As a consequence, anti-PTPRZ1 antibody administration significantly prolonged the survival of mice bearing the GSC xenografts (Fig. 7h, *P*<0.01, log-rank test). Collectively, these data demonstrate that the anti-PTPRZ1 antibody treatment is effective to inhibit GSC tumour growth.

To confirm that the antitumour effect of anti-PTPRZ1 antibody is exerted through blocking PTN–PTPRZ1 axis between GSC and TAMs, we constructed GBM xenografts co-implanted with GSCs (T4121) and MLCs, and administrated the tumour-bearing mice with anti-PTPRZ1 antibody (Supplementary Fig. 8a). The results demonstrated that co-implanted MLCs promoted GSC tumour growth, whereas the administration of anti-PTPRZ1 antibody impaired the pro-tumorigenic effect of MLCs on GSC xenografts (Supplementary Fig. 8b,c). Consequently, mice co-implanted with GSCs and MLCs had significantly shortened survival relative to those implanted with GSCs only (Supplementary Fig. 8d, *P*<0.01, log-rank test). However, treatment of anti-PTPRZ1 antibody compromised the oncogenic effect of MLCs on reducing animal survival (Supplementary Fig. 8d, *P*<0.01, log-rank test). Collectively, these data demonstrate that disrupting PTN–PTPRZ1 signalling by the anti-PTPRZ1 antibody impairs the supportive effect of TAMs, thus potently suppresses GSC tumour growth.

### PTN-PTPRZ1 axis activates Fyn-AKT pathway in GSCs

**Figure 8:**
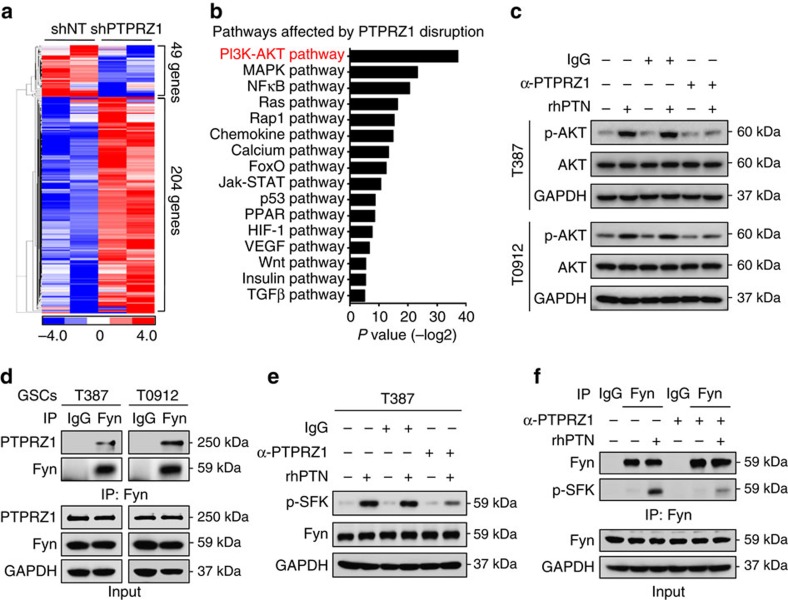
PTN–PTPRZ1 signalling activates the Fyn–AKT pathway in GSCs. (**a**,**b**) Hierarchical clustering analysis (**a**) and Kyoto Encyclopedia of Genes and Genomes pathway enrichment analysis (**b**) of differentially expressed genes between the PTPRZ1-silencing GSCs and control GSCs (fold change ≥2.0). Cluster analyses were performed and visualized using the Cluster/Java Treeview. Pathway enrichment significance is presented as a *P* value with log2 transformation, Fisher's exact test and *χ*^2^-test. (**c**) Immunoblot analyses of phospho-AKT (p-Ser473) and total AKT in GSCs (T387 and T0912), showing that rhPTN stimulation markedly increases AKT-activating phosphorylation, while the anti-PTPRZ1 antibody treatment compromises rhPTN-stimulated AKT activation in GSCs. Cells were pretreated with anti-PTPRZ1 antibody or control IgG for 1 h followed by rhPTN treatment for 20 min. (**d**) Co-immunoprecipitation of PTPRZ1 with the Fyn-specific antibody from T387 and T0912 GSC cell lysates. Precipitation with normal rabbit IgG was used as a negative control. PTPRZ1 binds to Fyn in GSCs. (**e**) Immunoblot analyses of p-SFK (Tyr416) and Fyn in T387 GSCs, showing that rhPTN stimulation markedly increases activating phosphorylation of SFK (p-Tyr416), while the anti-PTPRZ1 antibody treatment largely abrogates SFK activation in GSCs. (**f**) Co-immunoprecipitation of p-SFK (p-Tyr416) with the Fyn-specific antibody in T387 GSCs. Phosphorylated Fyn, as represented by immunoprecipitated p-SFK with Fyn antibody, was increased after rhPTN exposure, and was compromised by anti-PTPRZ1 antibody. Precipitation with rabbit IgG was used as a negative control.

To determine the downstream effectors mediating the PTN–PTPRZ1 signalling in GSCs, we performed gene-profiling analyses on GSCs expressing shPTPRZ1 or shNT control (Fig. 8a). The pathway enrichment analyses demonstrated that PI3K–AKT pathway, which is crucial for GSC maintenance^45^,^46^, was most significantly affected by PTPRZ1 disruption (Fig. 8b). Moreover, rhPTN stimulation potently elevated AKT phosphorylation (p-S473), whereas anti-PTPRZ1 treatment largely compromised rhPTN-induced AKT activation (Fig. 8c). These data demonstrate that PTN–PTPRZ1 signalling may promote GSC maintenance via activating PI3K–AKT pathway. To determine the signalling transducers between PTPRZ1 and AKT pathway, we examined the previously reported downstream targets of PTPRZ1 in GSCs and matched NSTCs^29^,^31^. The qRT–PCR results showed that the Src family member Fyn, also an upstream mediator of AKT pathway^47^,^48^, was preferentially expressed in GSCs relative to NSTCs (Supplementary Fig. 9a). Co-immunoprecipitation assays showed that Fyn interacted with PTPRZ1 (Fig. 8d), indicating that Fyn could be a downstream target of PTPRZ1 in GSCs. We then determined the effect of PTN–PTPRZ1 signalling on Fyn phosphorylation. Since Fyn shares the same phosphorylation site Tyr416 with other Src family kinases (SFK), we examined phosphorylated SFK (p-Tyr416) and found that rhPTN stimulation markedly increased phospho-SFK (p-SFK), while anti-PTPRZ1 antibody treatment largely abrogated the rhPTN-stimulated p-SFK upregulation (Fig. 8e and Supplementary Fig. 9b). Importantly, phosphorylated Fyn (p-Tyr416), as represented by immunoprecipitated p-SFK with Fyn antibody, was increased after rhPTN stimulation and could be compromised by anti-PTPRZ1 antibody (Fig. 8f). These results demonstrate that the PTN–PTPRZ1 signalling regulates Fyn phosphorylation, thus activates AKT pathway for GSC maintenance.

## Discussion

GBM contains abundant tumour-supportive TAMs to promote malignant progression^17^,^18^. TAM infiltration is correlated with poor prognosis of GBM patients^7^. Our previous study demonstrated that GSCs preferentially secrete periostin to recruit monocyte-derived macrophages and maintain these macrophages as M2 TAMs to support GBM malignant progression^13^. It is important to further determine how TAMs impact GSC maintenance and exert their tumour-promoting effects in tumour microenvironment. In this study, we uncover that M2 TAMs preferentially secrete the cytokine PTN to impact GSCs through its receptor PTPRZ1 to promote GSC maintenance and tumour growth. The TAM-secreted PTN and the GSC-expressed PTPRZ1 constitute a critical paracrine signalling in GSC niche. Disrupting the PTN–PTPRZ1 signalling by shRNA or the antibody blockade against PTPRZ1 largely abrogates the tumour-supportive effects of PTN and suppresses GBM growth driven by the tumour-initiating GSCs. This study indicates that the PTN–PTPRZ1 paracrine signalling axis mediates the molecular interplay between TAMs and GSCs, and plays a critical role in supporting GBM malignant growth.

TAMs and GSCs are crucial cellular components in promoting tumour growth in GBMs. The reciprocal interaction between TAMs and GSCs is pivotal for GBM propagation, therapeutic resistance and tumour recurrence^18^,^49^. GSCs not only recruit TAMs through secreting a potent chemoattractant periostin but also maintain the recruited TAMs as tumour-supportive M2 macrophages^18^,^50^. On the other hand, tumour-supportive TAMs in the GSC niche play critical roles in maintaining GSC phenotype and thus facilitate GBM progression and therapeutic resistance^13^,^14^. Although high TAM infiltration in the GSC niches suggests an important role of TAMs in GSC maintenance, the molecular mechanisms underlying the supportive functions of TAMs in promoting GSC maintenance and malignant progression in GBM have not been reported. Our study uncovered that CD11b^+^/CD163^+^ M2 TAMs promote GSC maintenance and GSC-driven tumour growth through the PTN–PRPTZ1 signalling, demonstrating that PTN is a critical molecular mediator bridging TAMs and GSCs in GBM tumour microenvironment. In addition, tumour-supportive TAMs may help cancer cells to escape from immune surveillance^10^. It has been shown that TAMs may prevent glioma cells including GSCs from being attacked by natural killers or adaptive T cells in GBMs^51^. Moreover, ablation of M2 TAMs inhibited GSC-driven tumour progression^14^, indicating that TAM engagement in the GSC niche is essential for maintaining GSC properties. Further investigations on the dynamic interplay between GSCs and TAMs at both cellular and molecular levels are crucial to enlarge our understanding of TAM functions in regulating GSC niche and immune microenvironment, which may help to improve the immunotherapy against GBM.

Tumour immunotherapy has become an attractive strategy for tumour treatment^51^,^52^. Current tumour immunotherapeutic strategies include vaccinations, immunomodulatory therapies, immune cell therapies (T-cell engineering or adoptive T-cell transfer) and cytokine therapies^52^. While immunotherapies have shown promising clinical activities in other cancers^53^, effective immunotherapy for GBM remains a daunting challenge, which could partially attribute to the enrichment of TAMs and their suppressive function against the antitumour immunity^4^,^5^. Thus, targeting the infiltrated TAMs may represent an attractive strategy to improve GBM immunotherapy^4^,^8^. Previous preclinical studies have implemented two main targeting approaches to inhibit TAMs. The first approach is to suppress TAM recruitment by targeting tumour cell-produced chemoattractants. For example, disruption of the GSC-secreted periostin successfully mitigated TAM infiltration and extended animal survival in GSC-derived GBMs^13^. The second approach is to inhibit functions of M2 TAMs through blocking polarization-related pathways. It has been shown that the colony stimulating factor 1 receptor (CSF-1R) inhibitor suppresses the CSF-1R-mediated M2 TAM polarization and markedly retards GBM progression^14^. Our study suggests another potential strategy to compromise the tumour-supportive functions of TAMs by disrupting the TAM-paracrined PTN signalling. PTN is preferentially expressed in TAMs in GBMs, indicating that PTN is a TAM-associated therapeutic target. In addition, a significant elevated level of PTN has been detected in patient serum or cell culture medium of several tumours^27^,^54^,^55^, suggesting that targeting PTN may inhibit tumour growth and benefit patient outcomes^55^. Furthermore, since TAMs may contribute to tumour resistance to current chemotherapies^56^, targeting TAMs by blocking their key paracrine mediator PTN may synergize with conventional anti-GBM therapies to improve patient survival.

The TAM-secreted PTN functions as a critical paracrine cytokine to impact GSCs through its receptor PTPRZ1. Our study demonstrated that PTN is dominantly secreted by CD163^+^ TAMs (Fig. 1 and Supplementary Fig. 10a) and PTPRZ1 is preferentially expressed by GSCs (Fig. 3 and Supplementary Fig. 10b). The binding of PTN to PTPRZ1 increases phosphorylation of the downstream tyrosine substrates, thereby activating the signal pathways related to cell survival, adhesion and migration^29^. The elevated expressions of PTPRZ1 and PTN and their pro-tumoural roles have been demonstrated in various cancers^32^,^33^,^34^,^35^,^57^,^58^. In addition, the PTN–PTPRZ1 signalling has been shown to regulate multiple biological processes in stem cells and progenitor cells^44^,^59^,^60^. Our study indicated that the PTN–PTPRZ1 signalling also plays an essential role in the maintenance of cancer stem cells in GBMs. We revealed that the TAM-secreted PTN acts through PTPRZ1 on GSCs to promote the self-renewal and tumour propagation of GSCs. As anaplastic lymphoma receptor tyrosine kinase (ALK) has also been reported as a receptor of PTN^55^,^61^,^62^, we have examined ALK expression in GSCs to address whether ALK could mediate the supportive effect of PTN on GSCs. The quantitative PCR results demonstrated that ALK expression was not consistently elevated in GSCs relative to NSTCs (Supplementary Fig. 11a), suggesting that ALK may not be associated with GSC phenotype. In addition, we analysed whether the expression of ALK or PTPRZ1 was associated with infiltrating TAMs in GBMs. The correlation analyses demonstrated that the level of PTPRZ1, but not ALK, was positively correlated with the expression of TAM marker CD163 in GBMs (Supplementary Fig. 11b,c). These results suggested that PTPRZ1 rather than ALK is associated with GSC phenotypes, and the PTN–PTPRZ1 signalling plays a major role in mediating the crosstalk between GSCs and TAMs.

The binding of PTN and PTPRZ1 could inhibit the phosphatase function of PTPRZ1 (refs 28, 63), and further promotes the phosphorylation of Fyn, which has been reported as a substrate of PTPRZ1 in HeLa cells^31^. Although the Fyn–AKT is the main downstream pathway activated by the PTN–PTPRZ1 signalling in GSCs, PTPRZ1 functioning as an intrinsically active phosphatase with a broad spectrum of substrates may mediate multiple pathways and affect other cellular activities. The consequences of genetic ablation of PTPRZ1 may be more complicated than the physiological changes on PTN stimulation, and may disrupt the intercellular phosphorylation balance to break down cell homeostasis, which requires further investigation. In conclusion, our findings underscore the crucial role of the PTN–PTPRZ1 paracrine signalling as a molecular link to mediate the tumour-supportive effects of M2 TAMs on GSC maintenance and GBM malignant growth. Disruption of PTN–PTPRZ1 signalling potently inhibits GSC-driven tumour growth, indicating that therapeutic targeting of PTN–PTPRZ1 signalling may effectively improve treatment for GBMs and potentially other malignant tumours.

## Methods

### GBM tumour specimens

Human GBM surgical specimens were obtained from the Southwest Hospital, The Third Military Medical University (TMMU) in Chongqing (China), with informed consents from patients or their guardians under an approved institutional review board protocol. Histopathological diagnoses were made by at least two neuropathologists based on the World Health Organisation (WHO) classification. The pathological characteristics of GBM specimens were summarized in Supplementary Table 3. The fresh GBM specimens were dissociated followed by FACS to isolate GSCs and TAMs. The frozen GBM sections and the formalin-fixed, paraffin-embedded GBM sections were stored at −20 °C or at room temperature, respectively. All procedures were performed in accordance with the principles of the Helsinki Declaration and approved by the institutional ethics committees.

### Cell culture

GSCs and matched NSTCs were isolated from primary GBMs or patient-derived GBM xenografts (Southwest Hospital and Cleveland Clinic) through FACS as previously described^13^,^21^,^40^,^41^,^64^. Briefly, GBM tumours were dissociated with Papain Dissociation System (Worthington Biochemical). The isolated tumour cells were recovered in stem cell medium, that is, Neurobasal medium (Invitrogen) with B27 Supplement (20 μl ml^−1^, Life Technologies), epidermal growth factor (20 ng ml^−1^, PeproTech) and basic fibroblast growth factor (20 ng ml^−1^, PeproTech), for 6 h to re-express the GSC surface markers. Cells were then labelled with a fluorescein isothiocyanate (FITC)-conjugated anti-CD15 antibody (BD Biosciences, 347423) and a P-phycoerythrin (PE)-conjugated anti-CD133 antibody (Miltenyi, 130-090-854) at 4 °C for 40 min followed by FACS to isolate the GSCs (CD15^+^/CD133^+^) and NSTCs (CD15^−^/CD133^−^). The cancer stem cell characteristics of GSCs were validated by preferential expressions of GSC markers (SOX2, OLIG2, CD15 and CD133) and a series of functional assays, including the serial tumoursphere formation assay, the serum-induced differentiation assay and the *in vivo* limiting dilution assay, to confirm GSC self-renewal potential, multi-lineage differentiation potency and the tumour formation capacity as described previously^13^,^21^,^40^,^41^. The enriched GSCs were constantly maintained as GBM xenografts and were only dissociated, sorted and cultured in the stem cell medium *in vitro* for functional experiments, whereas the NSTCs were cultured in DMEM (Gibco) with 10% fetal bovine serum (FBS, Gibco). Cells have been authenticated by examining their karyotypes and morphologies. All cells have been tested for mycoplasma contamination by PCR and were verified to be mycoplasma-free.

### Isolation of CD11b^+^/CD163^+^ M2 TAMs and CD11b^+^/CD163^−^ TAMs

FACS was performed to obtain CD11b^+^/CD163^+^ M2 TAMs and the CD11b^+^/CD163^−^ control TAMs from human GBMs. Briefly, cells dissociated from human GBM tumours were incubated with Alexa Fluor 488-conjugated mouse anti-human CD11b (R&D Systems, FAB16991G-100) and mouse anti-human CD163-APC (Miltenyi, 130-100-612) or control IgG (Miltenyi, 130-098-846 and R&D Systems, IC0041G) for 30 min at 4 °C, followed by FACS using a BD FACSAria II cell sorter.

### U937 cells and the U937-derived M2 MLCs

U937 cells were purchased from American Type Culture Collection and were maintained in RPMI 1640 medium with 10% FBS (Gibco). U937-derived MLCs were used as a macrophage model to mimic TAM^13^,^37^,^38^. Briefly, U937 cells were polarized into macrophages using PMA treatment (Sigma, 5 nM), and then induced into M2-like macrophages using a combined treatment of IL-4, IL-10 and TGF-β (Peprotech, 20 ng ml^−1^) as described^39^. The M2 macrophage characteristics of the U937-derived MLCs were confirmed by the elevated expressions of the M2 markers (CD163, Fizz1, Arg1 and CD206) and the reduced expressions of the M1 markers (iNOS and MHC-II). Cells have been authenticated by STR profiling and have been verified to be mycoplasma-free.

### Immunofluorescent staining

Slides with formalin-fixed GBM tissues or GSCs were incubated with 10% of goat blocking serum (ZSGB-Bio, ZLI-9022) for 1 h, then with diluted primary antibodies at 4°C, overnight. Slides were further incubated with fluorophore-conjugated secondary antibody for 30 min. The cell nuclei were counterstained with the 4,6-diamidino-2-phenylindolefor 15 min. Images were obtained using a Leica SP5 spectral confocal microscope or an AMG EVOS FL microscope. Primary antibodies listed as follows: anti-PTN (Proteintechs, 10821-1-AP, 1: 100); anti-CD163 (Santa Cruz, sc-33715 and sc-33560, 1: 50); anti-Iba1 (Abcam, ab5076, 1: 50); anti-PTPRZ1 (BD Biosciences, 610179, 1: 200); anti-SOX2 (Cell Signaling, #3579, 1: 200); anti-OLIG2 (Millipore, MABN50-KC, 1: 100); anti-CD133 (Millipore, MAB4399, 1: 100); and anti-mCherry (Abcam, ab167453, 1:100). To determine PTN expression in TAM^high^ or TAM^low^ tumour regions, GBM specimens were co-stained with PTN and the TAM marker Iba1 or the M2 TAM marker CD163. For each specimen, the PTN expression was calculated under five randomly selected microscopic fields at × 400 by ImageJ software. To separate Iba1^high^ from Iba1^low^ and CD163^high^ from CD163^low^ areas of the tumours, five randomly selected areas (1,360 × 1,024 dots per inch) from each specimen were calculated for the average signal density of Iba1 or CD163 by ImageJ software. Within a given tumour, the areas with an Iba1 signal density lower than the average level were defined as Iba1^low^ areas; otherwise, the areas were classified as Iba1^high^ areas. CD163^high/low^ areas were defined by the same approach. All immunofluorescent stainings were independently repeated for at least three times.

### Immunohistochemistry

IHC and analyses were performed according to the manufacturer's guidelines of Dako REAL EnVision Detection System (DAKO). Primary antibodies used for IHC included anti-CD163 (ZSGB-Bio, ZM-0428, without dilution), anti-PTN (Proteintechs, 10821-1-AP, 1: 100), anti-SOX2 (Cell Signaling, #3579, 1: 200), anti-PTPRZ1 (BD Biosciences, 610179, 1: 200), anti-Ki67 (ZSGB-Bio, TA500265, 1: 50) and GFAP (Maixin-Bio, Kit-0031, without dilution). The images were obtained under a BX51 microscope (Olympus) equipped with a DP72 digital camera (Olympus). The staining signal of PTPRZ1 in GBM xenografts was scored according to the proportion of positive cells and the staining intensity as described previously^65^. The percentages of CD163-, PTN- and SOX2-positive cells in human GBMs and Ki67 proliferation index in GBM xenografts were quantified under × 400 magnification of microscopic field in five randomly selected areas for each tumour specimen.

### Cell viability assay and *in vitro* limiting dilution assay

Cell viability assay was conducted using Cell Titer-Glo Luminescent Cell Viability Assay kit (Promega) according to the manufacturer's guidance. For *in vitro* limiting dilution assay, GSCs were implanted into a 96-well plate at a gradient of 1, 5, 10, 20, 40 or 80 cells per well, with 10 replicates for each gradient. The number of tumourspheres in each well was determined after incubation for 7 days, and the sphere formation efficiency were calculated using the Extreme Limiting Dilution Analysis (http://bioinf.wehi.edu.au/software/elda)^66^.

### Tumoursphere formation assay

GSCs were plated in 96-well plates at a density of 1,000 cells per well and tumoursphere numbers and sizes were calculated at the seventh day after cell placement^40^.

### GSC differentiation assay

GSC differentiation was induced via withdrawing growth factors and adding serum^40^. Briefly, GSCs were induced to differentiate in the serum-containing medium (DMEM with 10% FBS). At indicated time points, cells were collected to examine the expressions of PTPRZ1, the GSC marker SOX2 and the differentiation markers GFAP and MAP2 using immunoblot assay or immunofluorescent staining.

### Lentiviral vector construction

The human PTN and the PTPRZ1-specific shRNA vectors and the shNT vectors were purchased from Sigma and Obio Technology Corp. Ltd., China, respectively, with the shRNA sequences listed in Supplementary Table 4. The lentivirus packaging and transduction were conducted as described previously^40^,^41^. Cells stably expressing shPTN or shPTPRZ1 were enriched by puromycin selection for positive clones.

### Quantitative real-time PCR

qRT–PCR was performed on a CFX96 Real-Time PCR Detection System (Bio-Rad). The sequences of primers used in this study were listed in Supplementary Table 5. The expression of *ACTB* (gene encoding for β-actin) and *GAPDH* were used for the normalization.

### Co-immunoprecipitation and immunoblot assay

For co-immunoprecipitation assay, collected cells were lysed in the lysis buffer containing 50 mM Tris-HCl (pH 7.4), 150 mM NaCl, 5 mM EDTA, 10% glycerol, 1% NP-40, 1 mM sodium fluoride and 1 mM sodium orthovanadate. Insoluble proteins (500 μg per sample) were incubated with anti-Fyn antibody (Santa Cruz, sc-16) and Protein A/G Plus Agarose beads (Santa Cruz) for 14 h at 4°C with constant rotation. Immunocomplexes were washed four times with 0.1% Triton-100 in PBS and eluted by boiling in the Laemmli buffer. Primary antibodies used for immunoblot were listed as follows: anti-PTPRZ1 (BD Biosciences, 610179, 1: 1,000); anti-SOX2 (Bethyl Laboratories, A301-739A, 1: 1,000); anti-Phospho-AKT (p-Ser473, Cell Signaling, #4060, 1: 2,000); anti-AKT (Cell Signaling, #2966, 1: 500); anti-CD133 (Millipore, MAB4399, 1: 500); anti-GFAP (Covance, PRB-571C, 1: 1,000); anti-MAP2 (Covance, SMI-52R, 1: 1,000); anti-p-SFK (Tyr416, Cell Signaling, #6943, 1: 500); anti-Fyn (Cell Signaling, #9320, 1: 500); anti-Tubulin (Sigma, T9026, 1: 10,000); and anti-GAPDH (Cell Signaling, #2118, 1: 2,000). Representative uncropped immunoblots were presented in Supplementary Fig. 12. All immunoblot results were independently repeated for at least three times.

### Flow cytometry analyses

To determine the expression of PTPRZ1 in glioma cells, the indicated tumour cells were incubated with anti-human PTPRZ1 antibody (R&D Systems, MAB26881) or with the isotype IgG (R&D Systems, #54447) at 4°C for 1 h. Cells were further incubated with fluorochrome-labelled secondary antibody (Invitrogen) followed by flow cytometry analyses using a BD FACSAria II cell sorter. Data were analysed and presented using FlowJo software (Tree Star). To isolate PTPRZ1^+^ and PTPRZ1^−^ glioma cells from GBM xenografts, tumours were disaggregated using Papain Dissociation System (Worthington Biochemical) according to the manufacturer's instructions. Briefly, the tissues were mechanically dissociated by cutting into small pieces and were digested using the enzyme mixture solution containing DNase and Papain enzyme at 37 °C. The tissue was further mechanically dissociated and the suspension was filtered with a 70 μm cell strainer (BD Biosciences) to remove tissue pieces and the myelin in mouse brains. The mixture was used for density gradient centrifugation using Percoll (Solarbio, P8370) to isolate the cell part at the intermediate layer and to further remove mouse myelin at the top layer. Cells were then cultured in Neurobasal medium (Invitrogen) with B27 Supplement and growth factors for 6 h to recover surface antigens, then passed through a 30 μm nylon mesh to obtain a single-cell suspension. Human tumour cells were enriched using Mouse Cell Depletion Kit (Miltenyi, 130-104-694) and incubated with rat anti-human PTPRZ1 antibody (R&D Systems) or the isotype IgG (R&D Systems) to sort for PTPRZ1^+^ and PTPRZ1^−^ glioma cells.

### Orthotopic xenografts and *in vivo* limiting dilution assay

Intracranial transplantation of GBM cells was performed to establish orthotopic xenografts in mice^65^. T4121 GSCs were isolated from the xenografts derived from an adult GBM (WHO grade IV) from a 53-year-old patient^40^. T0912 GSCs were isolated from the xenografts derived from an adult GBM (WHO grade IV) from a 35-year-old male patient^42^. Male non-obese diabetic/severe combined immunodeficiency (NOD/SCID) mice of 4–6 weeks were purchased from and maintained in Laboratory Animal Centre at Southwest Hospital. No randomization was used for the *in vivo* experiments. To determine the role of PTN in mediating the tumour-promoting effect of TAMs on GSC-driven tumour growth, U937-derived MLCs expressing mCherry and shPTNs or shNT were co-implanted in mouse brains with T4121 GSCs (5 × 10^3^ cells per mouse). Tumour growth was monitored via bioluminescent imaging using IVIS Spectrum system and quantified by Living Image Software. To determine the effect of PTPRZ1 disruption on GSC-driven tumour growth, T4121 GSCs (2 × 10^4^ cells per mouse) or T0912 GSCs (5 × 10^4^ cells per mouse) expressing shPTPRZ1 or shNT were implanted and tumour growth was monitored by bioluminescent imaging. To determine the effect of TAMs on the growth of PTPRZ1^+^ and PTPRZ1^−^ tumours, the PTPRZ1^+^ and PTPRZ1^−^ glioma cells (T4121) were isolated from GBM xenografts through FACS and were co-implanted with U937-derived MLCs to construct GBM xenografts. For *in vivo* limiting dilution assays, orthotopic xenografts were constructed using FACS-sorted PTPRZ1^+^ and PTPRZ1^−^ cells (T0912) that were implanted at the gradient of 5 × 10^2^, 5 × 10^3^ and 5 × 10^4^ cells per mouse (*n*=5 per group). Tumour growth was monitored by bioluminescent imaging and the tumour formation incidence was calculated using the Extreme Limiting Dilution Analysis (http://bioinf.wehi.edu.au/software/elda/). All animal experiments were carried out in accordance with the Guide for the Care and Use of Laboratory Animals and approved by the Institutional Animal Care and Use Committee of Southwest Hospital, TMMU.

### Treatment with anti-PTPRZ1 antibody

To determine the therapeutic value of disrupting PTN–PTPRZ1 signalling via anti-PTPRZ1 antibody treatment, T0912 GSCs (1 × 10^5^ cells per mouse) were pre-incubated with anti-PTPRZ1 antibody (5 μg ml^−1^; R&D Systems, MAB26881) or the isotype control IgG (R&D Systems, #54447) for 72 h followed by intracranial implantation. After injection, mice were intravenously treated with anti-PTPRZ1 antibody (2 mg kg^−1^; R&D Systems, MAB26881) or isotype IgG (2 mg kg^−1^; R&D Systems, #54447) twice per week until moribund. Tumour growth was monitored by bioluminescent imaging and mice were killed when neurological signs occurred. All *in vivo* experiments were carried out in accordance with the Guide for the Care and Use of Laboratory Animals and approved by the Institutional Animal Care and Use Committee of Southwest Hospital, TMMU.

### Microarray analysis from the TCGA database

The gene expression in human GBMs and patient survival information were analysed using gene-profiling data (Agilent-4502A microarrays) from the TCGA database (https://tcga-data.nci.nih.gov/tcga).

### Microarray analysis from GEO database

Microarray data of GEO (http://www.ncbi.nlm.nih.gov/geo), GSE37475, GDS3885 and GSE54791, were enrolled in this study. GSE37475 profile contained gene expression data of M2^low^ TAMs and M2^high^ TAMs isolated from murine gliomas^14^. The differentially expressed molecules (fold change ≥2.0) between M2^high^ and M2^low^ populations were overlapped with a full list of mouse-secreted proteins from the Secreted Protein Database^36^, to identify the soluble factors whose expressions were reduced in M2^low^ TAMs relative to the M2^high^ TAMs. GDS3885 profile included microarray data from 27 panels of GSCs and 36 panels of NSTCs^67^. GSE54791 data set contained gene-profiling data from 3 pairs of GSCs and NSTCs^68^. Gene cluster analyses were performed and visualized using Cluster/Java Treeview^69^. Candidate genes enriched in GSCs were identified when the average gene expression ratio of GSCs/NSTCs was ≥3.0-fold. Gene expression profiles of GSCs expressing shRNAs against PTPRZ1 (shPTPRZ1) and control shNT (GEO: GSE69081) were performed using the Affymetrix GeneChip Human Genome U133 Plus 2.0 Array and the PrimeView Human Gene Expression Array (CapitalBio Technology). All procedures were carried out based on the manufacturer's guideline. Differentially expressed genes were identified when gene expression ratio of shPTPRZ1/shNT was ≥2.0- or ≤0.5-fold. Cluster analyses were performed and visualized using Cluster/Java Treeview.

### Pathway enrichment analyses

Kyoto Encyclopedia of Genes and Genomes pathway enrichment analyses of differentially expressed genes between shPTPRZ1-expressing GSCs and GSCs expressing shNT were performed by Genminix Informatics Ltd. Fisher's exact test and *χ*^2^-test were used to calculate the statistical significance, which was defined as *P*<0.05.

### Statistical analyses

No statistical method was used to predetermine sample size. The investigators were not blinded to allocation during experiments and outcome assessment. All data were included for statistical analyses using PASW Statistics 18 or GraphPad Prism 6.0. Unpaired Student's *t*-test (two-tailed) was used for the comparison between unpaired two-groups and one-way ANOVA was applied for multi-group data comparison. The variance was similar between the groups that were being statistically compared. Bivariate correlation analysis (Pearson's *r* test) was used to examine the correlation of two variables in human specimens. All data met the assumptions of the tests. Survival estimates were calculated using the Kaplan–Meier analysis, with cut-off point generated by X-tile software (Yale University)^70^. Briefly, the expressions of target genes and patient survival information from the TCGA database were loaded into X-Tile as tab-delimited text file. By running ‘Kaplan–Meier' programme, the cohort was then divided into two data sets with the optimal cut points generated according to the highest *χ*^2^-value defined by log-rank test and Kaplan–Meier analyses. Bar graphs were presented as means±s.d. or means±s.e.m., with statistical significance at *P<0.05 or **P<0.01.

### Data availability

The microarray data (GSE37475, GDS3885 and GSE54791) referenced in the study are available in a public repository from the GEO website (http://www.ncbi.nlm.nih.gov/geo). The authors declare that all the other data supporting the findings of this study are available within the article and its Supplementary Information files and from the corresponding authors on reasonable request.

## Additional information

**How to cite this article:** Shi, Y. *et al*. Tumour-associated macrophages secrete pleiotrophin to promote PTPRZ1 signalling in glioblastoma stem cells for tumour growth. *Nat. Commun.* **8,** 15080 doi: 10.1038/ncomms15080 (2017).

**Publisher's note:** Springer Nature remains neutral with regard to jurisdictional claims in published maps and institutional affiliations.

## Supplementary Material

Supplementary Information Supplementary Figures and Supplementary Tables

Supplementary Data 1 Differential expressed genes between M2 subtype-low TAMs and naive TAMs isolated from murine gliomas (GEO database, GSE37475).
