# Supplementary material for: Tumour-associated macrophages secrete pleiotrophin to promote PTPRZ1 signalling in glioblastoma stem cells for tumour growth
Source: Nat Commun. 2017 Jun 1;8:15080. doi: 10.1038/ncomms15080 (PMC5461490; doi:10.1038/ncomms15080)
Supplement: Supplementary Information — Supplementary Figures and Supplementary Tables [file ncomms15080-s1.pdf]

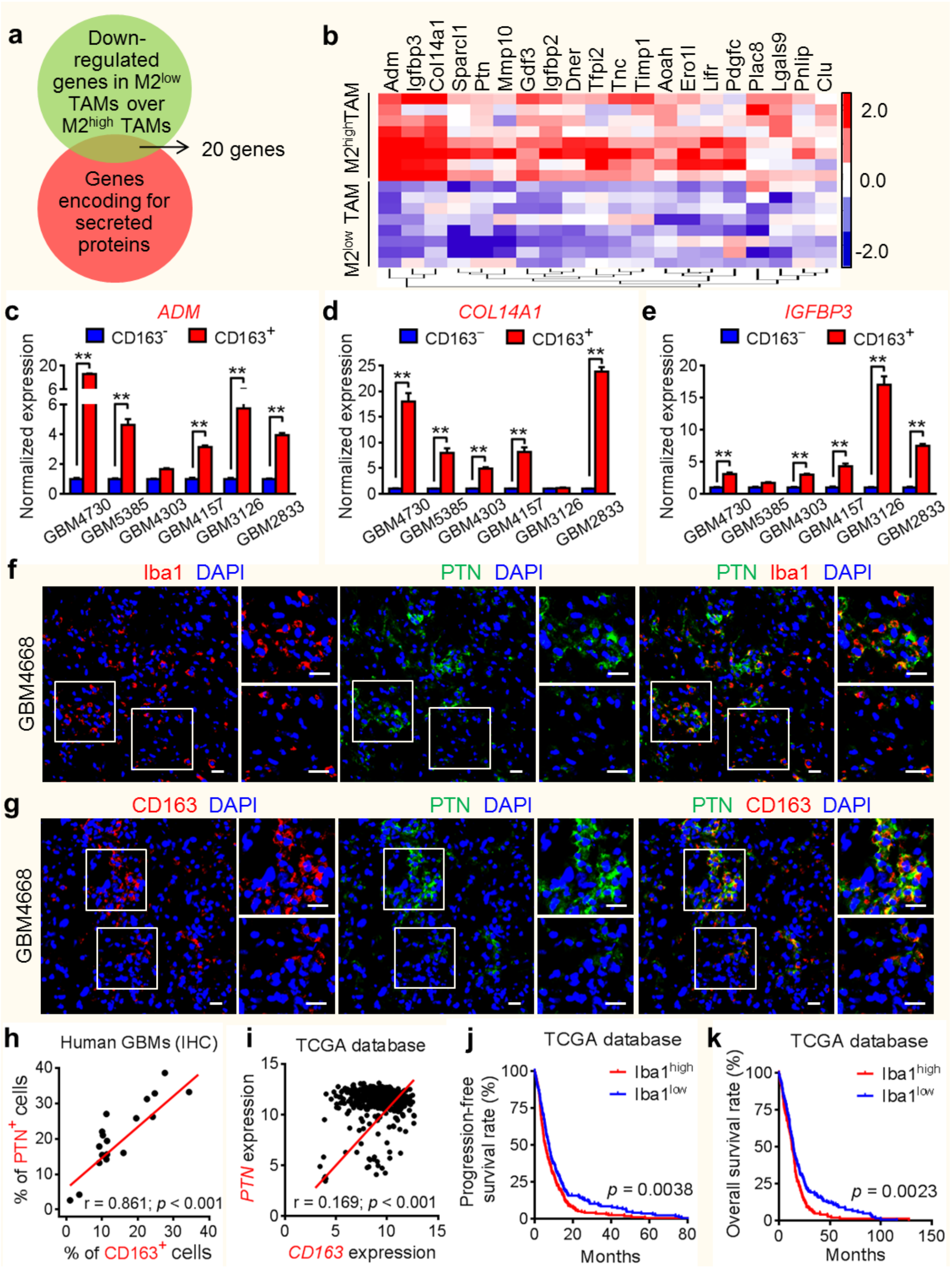


**Supplementary Figure 1.** PTN is co-enriched with CD163$^+$ M2 TAMs in human GBMs.

(**a**) Venn diagram showing gene candidates with reduced expressions in M2$^{low}$ TAMs (GEO, GSE37475) and overlapped with those encoding for secreted proteins (Secreted Protein Database, http://spd.cbi.pku.edu.cn/). M2$^{low}$ TAMs were induced by the treatment of CSF-1 receptor inhibitor BLZ945 to suppress M2 polarization and were isolated from murine gliomas.

(**b**) Heatmap of the secreted protein-coding genes with reduced expression in M2$^{low}$

TAMs relative to M2$^{high}$ TAMs identified from (**a)**.

(**c-e**) qRT-PCR analyses of the expression of *ADM* (**c**), *COL14A1* (**d**), or *IGFBP3* (**e**) between FACS-sorted CD11b$^{+}$/CD163$^{+}$ M2 TAMs and CD11b$^{+}$/CD163$^{-}$ control TAMs from 6 cases of human GBMs. Data are shown as means ± s.e.m., $^{**}p < 0.01$, student's *t*-test.

(**f-g**) Immunofluorescent staining of PTN (in green) and the TAM marker Iba1 (in red, **f**) or the M2 TAM marker CD163 (in red, **g**) in human GBMs. Scale bar represents 25 μm.

(**h-i**) Bivariate correlation analyses of the expressions of PTN and M2 TAM marker CD163 in human GBMs as examined by IHC staining (**h**) or by bioinformatic analyses of the gene profiling data from the TCGA database (**i**). $p < 0.001$, Pearson *r* test.

(**j-k**) Kaplan-Meier survival analysis of Iba1 expression and the progression-free survival (**j**) or overall survival (**k**) of GBM patients from the TCGA database. The cut-off point for stratification was generated using X-tile software according to Iba1 level. $p = 0.0038$ (**j**), $p = 0.0023$ (**k**), log-rank test.

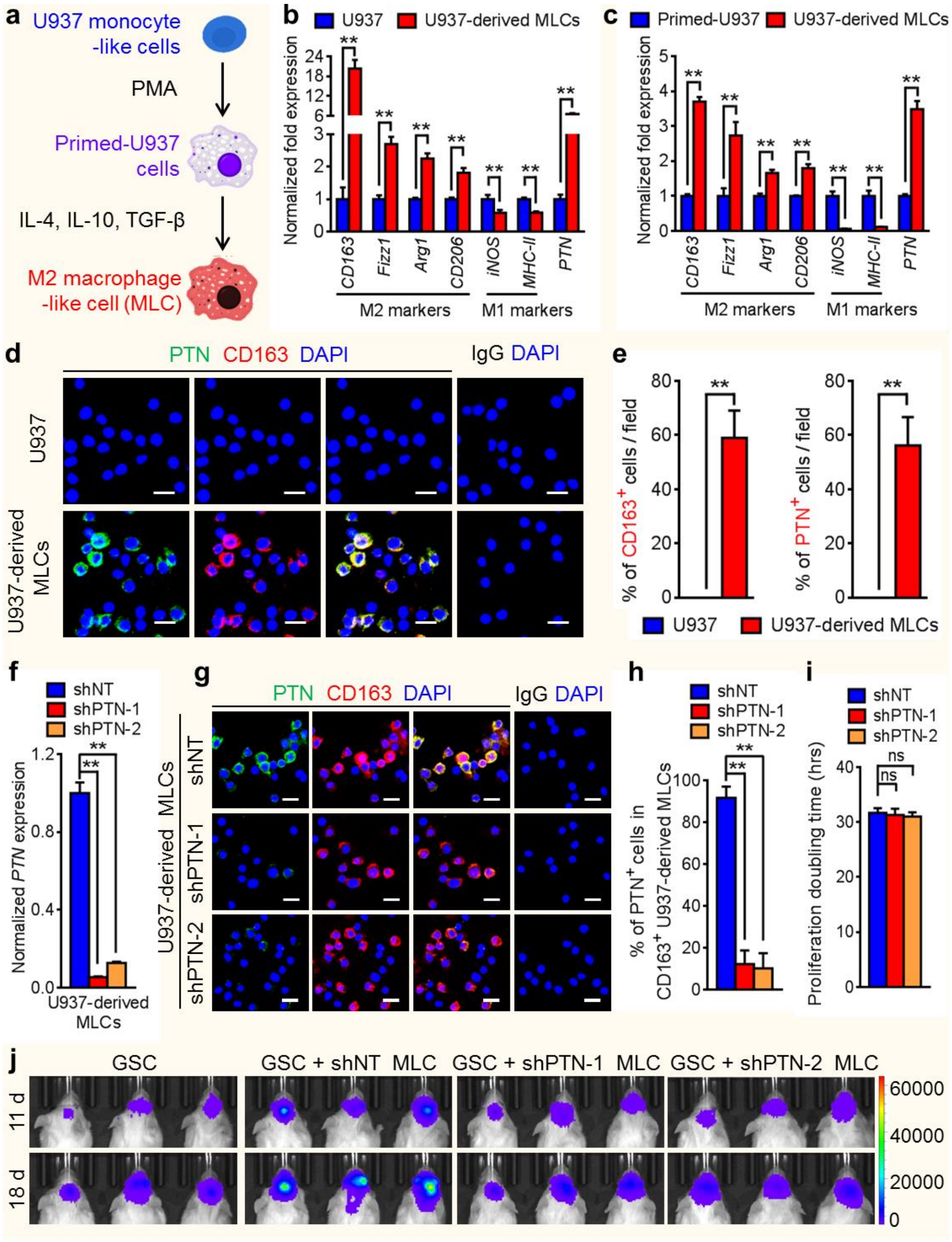


**Supplementary Figure 2.** U937-derived MLCs harbor M2 TAM properties and recapitulate the tumour-supportive functions of M2 TAMs *in vivo*.

(**a**) A schematic diagram showing the polarization of U937 monocytes into M2 macrophage-like cells (MLCs).

(**b-c**) qRT-PCR analyses of the expressions of M2 markers (*CD163*, *Fizz1*, *Arg1* and *CD206*), M1 markers (*iNOS* and *MHC-II*) and *PTN* between U937 and the U937-derived MLCs (**b**) or between primed-U937 and the U937-derived MLCs (**c**). Data are shown as means ± s.e.m., $**p < 0.01$, student's *t*-test.

(**d-e**) Immunofluorescent staining (**d**) of PTN (in green) and the M2 TAM marker CD163 (in red) in U937 and the U937-derived MLCs. The quantification of $CD163^+$ cells (**e**, left panel) or $PTN^+$ cells (**e**, right panel) is shown. Scale bar represents 20 μm. Data are shown as means ± s.d., $^{**}p < 0.01$, student's *t*-test.

(**f-h**) qRT-PCR analysis (**f**) and immunofluorescent staining (**g, h**) of PTN expression in the MLCs expressing shPTN or shNT control. Scale bar represents 20 μm. Data are shown as means ± s.d., $^{**}p < 0.01$, ANOVA test.

(**i**) The doubling time of the U937-derived MLCs expressing shPTN or shNT control. Data are shown as means ± s.d., ns, not significant, ANOVA test. Hrs, hours.

(**j**) The bioluminescent images of intracranial GBM xenografts implanted with GSCs only or co-implanted with GSCs and MLCs expressing shPTN or shNT at the indicated time points.

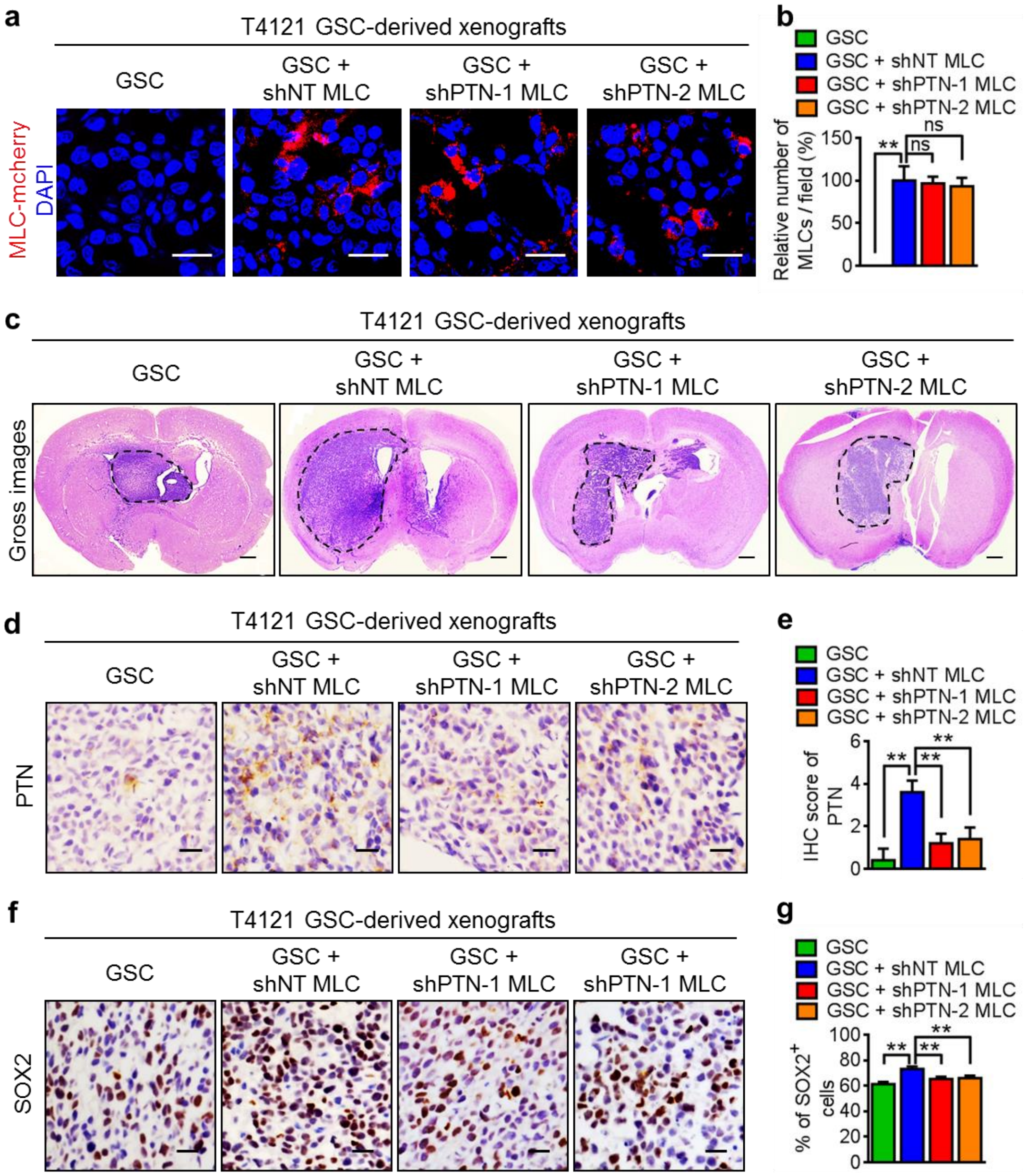

**Supplementary Figure 3.** U937-derived MLCs promote GSC tumour growth and increase the level of PTN in GSC xenografts.

(**a-b**) Representative immunofluorescent images of mCherry-labelled MLCs (**a**, in red) and the relative number of mCherry$^{+}$ MLCs (**b**) in GBM xenografts implanted with GSCs only or co-implanted with GSCs and MLCs expressing shPTN or shNT. The number of mCherry$^{+}$ MLCs of each group relative to the GSC + shNT MLC group was presented. Scale bar represents 25 μm. Data are shown as means ± s.d., $**p < 0.01$, ns, not significant, ANOVA test.

(**c**) Hematoxylin and eosin (H&E) staining of the cross sections of mouse brains bearing xenografts derived from GSC, GSC + shNT MLC or GSC + shPTNs MLC. The dashed line indicated the xenograft regions in mouse brain. Scale bar represents 500 μm.

(**d-e**) Representative IHC images (**d**) and the quantification (**e**) of PTN in GBM xenografts derived from GSC, GSC + shNT MLC or GSC + shPTNs MLC. Scale bar represents 25 μm. Data are shown as means ± s.d., $**p < 0.01$, ANOVA test.

(**f-g**) Representative IHC images of SOX2 (**f**) and the percentage of GSCs marked by SOX2 (**g**) in xenografts derived from GSC, GSC + shNT MLC or GSC + shPTNs MLC. Scale bar represents 25 μm. Data are shown as means ± s.d., $**p < 0.01$, ANOVA test.

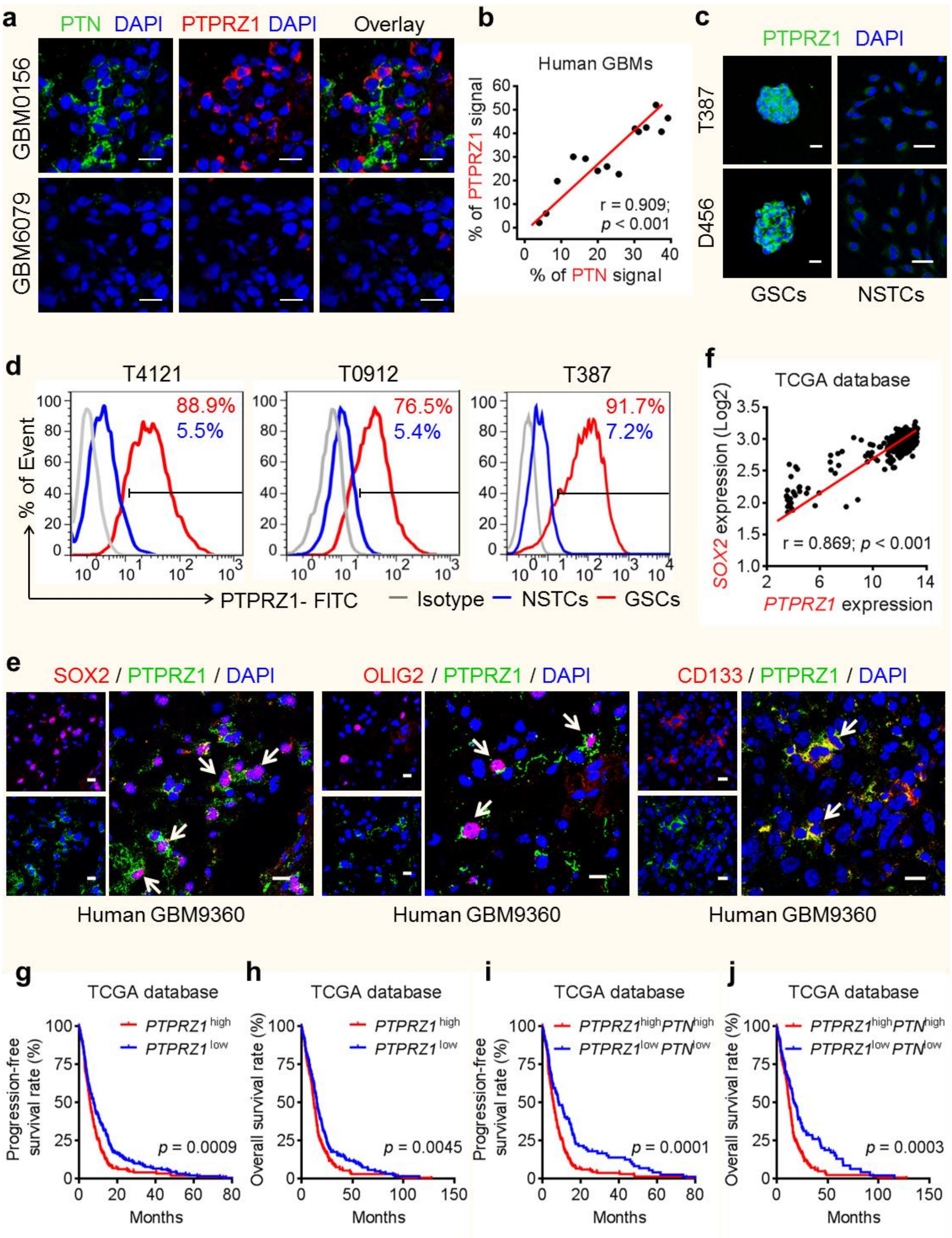


**Supplementary Figure 4.** PTPRZ1 is preferentially expressed in GSCs and combines with PTN to predict poor prognosis of patients with GBM.

(**a**) Representative immunofluorescent stainings of PTN (in green) and PTPRZ1 (in red) in human GBMs. Scale bar represents 20 μm.

(**b**) Bivariate correlation analyses showing a positive correlation of PTN and PTPRZ1 expressions as examined by immunofluorescent stainings shown in (**a**) in human GBMs. $p < 0.001$, Pearson $r$ test.

(**c**) Immunofluorescence of PTPRZ1 (in green) in GSC tumourspheres and NSTCs

isolated from T387 and D456 human GBM xenografts. Scale bar represents 25 μm.

(**d**) Flow cytometry analysis of PTPRZ1 expression in GSCs and matched NSTCs from T4121, T0912 and T387 GBMs.

(**e**) Co-immunofluorescent stainings of PTPRZ1 (in green) and the GSC marker SOX2 (in red, left panel), OLIG2 (in red, middle panel) or CD133 (in red, right panel) in human GBMs. Scale bar represents 25 μm.

(**f**) Bivariate correlation analyses showing a positive correlation of PTPRZ1 and the GSC marker SOX2 in 541 cases of human GBMs from the TCGA database. $p < 0.001$, Pearson $r$ test.

(**g**, **h**) Kaplan-Meier survival analysis of PTPRZ1 expression and the progression-free survival (**g**) or overall survival (**h**) of patients with GBM from the TCGA database. The cut-off point for stratification was generated using X-tile software according to the PTPRZ1 level. Log-rank test.

(**i**, **j**) Kaplan-Meier survival analyses of the combined PTPRZ1 and PTN expressions and the progression-free survival (**i**) or overall survival (**j**) of patients with GBM from the TCGA database. The cut-off point for stratification was generated using X-tile software according to the PTPRZ1 and PTN levels. Log-rank test.

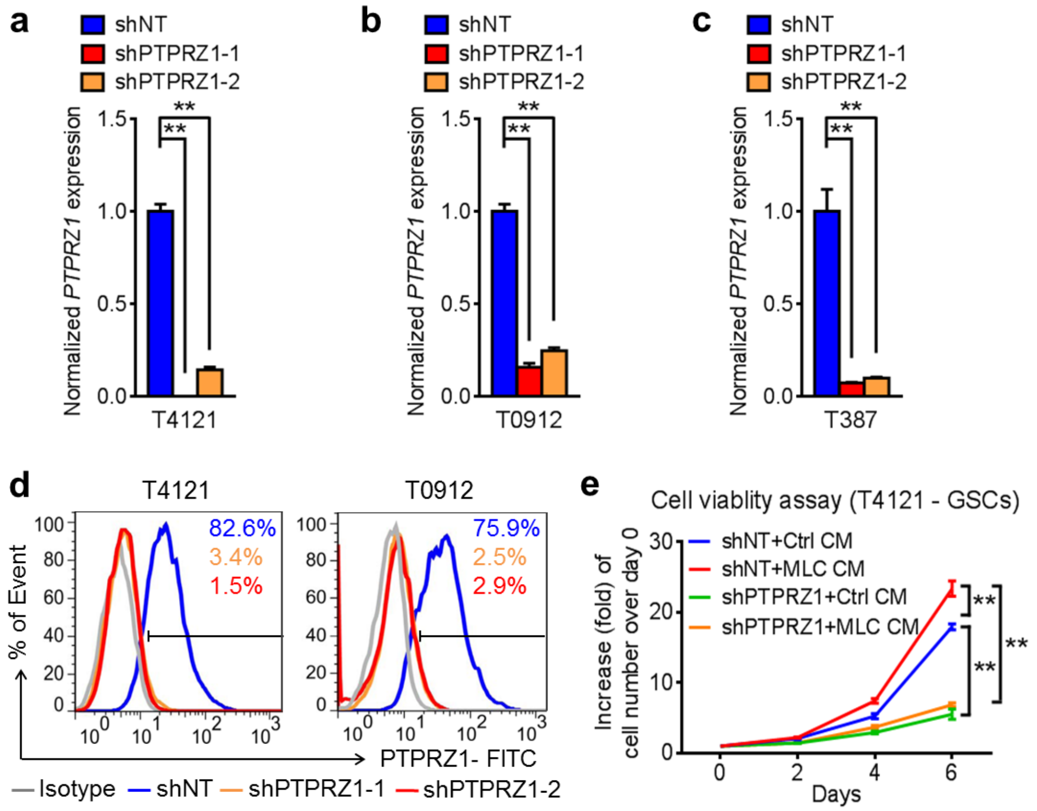


**Supplementary Figure 5.** ShRNAs against PTPRZ1 reduce PTPRZ1 expression in GSCs and compromise the tumour-supportive role of TAMs on GSC proliferation

(**a-c**) qRT-PCR analyses showing the level of *PTPRZ1* in GSCs expressing

shPTPRZ1 was significantly lower than the GSCs expressing the shNT control. GSCs were derived from T4121 (**a**), T0912 (**b**) or T387 (**c**) human GBM xenografts. Data are shown as means ± s.e.m., ** $p < 0.01$, ANOVA test.

(**d**) Flow cytometry analysis of PTPRZ1 expression in T4121 and T0912 GSCs expressing shPTPRZ1 or the shNT control.

(**e**) Cell viability assay of GSCs cultured in the conditioned medium of MLCs. Data are shown as means ± s.d., ** $p < 0.01$, ANOVA test.

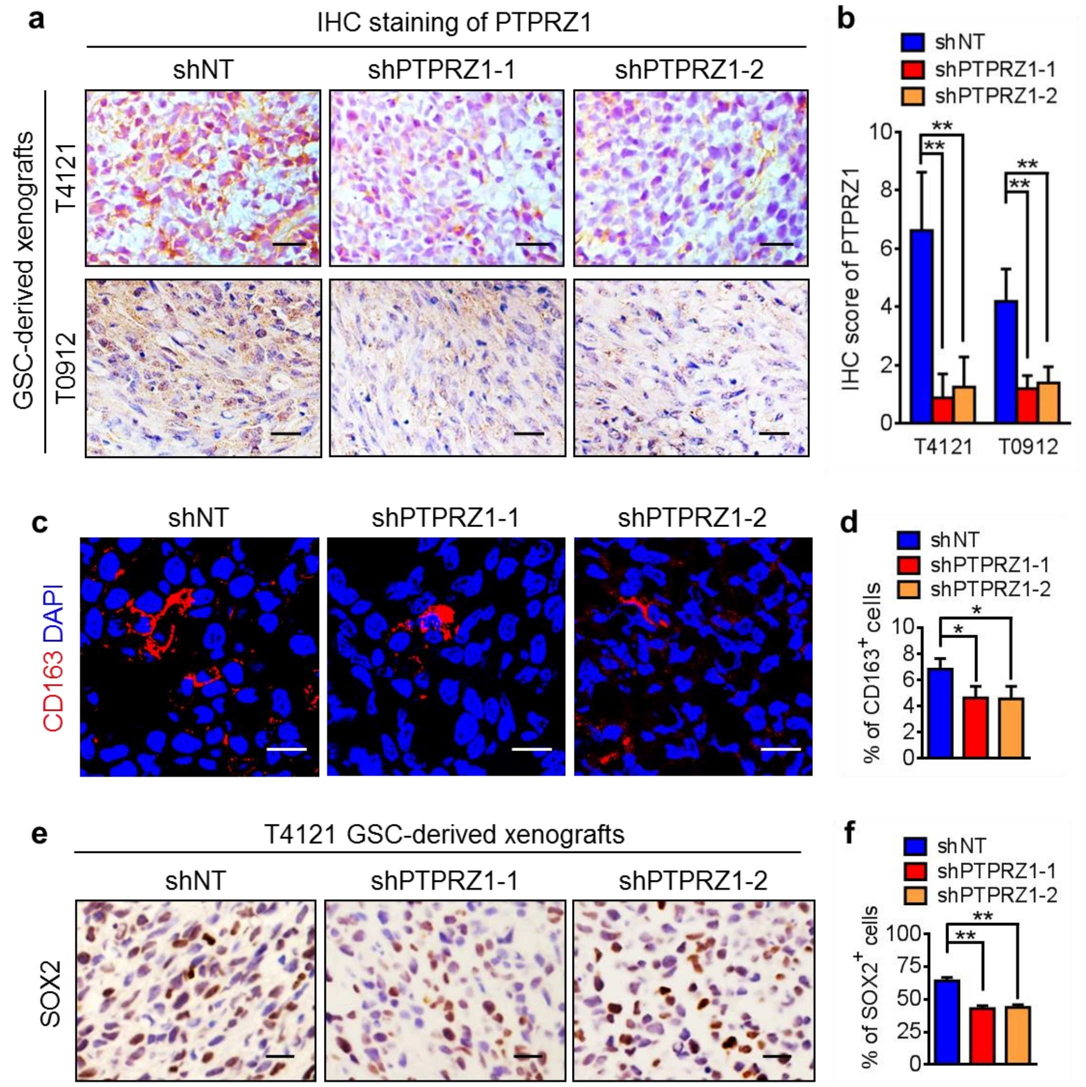


**Supplementary Figure 6.** Disrupting PTPRZ1 expression in GSCs impairs TAM infiltration, reduces GSC proportion in GSC xenografts.

(**a, b**) Representative IHC images (**a**) and the quantification (**b**) of PTPRZ1 expression in xenografts derived from T4121 GSCs (upper panels) or T0912 GSCs (lower panels) expressing shPTPRZ1 or shNT control. PTPRZ1 expression was scored according to the proportion of positive tumour cells and the staining intensity. Data are shown as means ± s.d., ** $p < 0.01$, ANOVA test. Scale bar represents 25 μm.

(**c, d**) Representative immunofluorescent images (**c**) and the percentage (**d**) of

infiltrating TAMs marked by CD163 in xenografts derived from T4121 GSCs expressing shPTPRZ1 or shNT. Data are shown as means ± s.d., *$p$ < 0.05, ANOVA test. Scale bar represents 25 μm.

(**e, f**) Representative IHC images of SOX2 (**e**) and the percentage of GSCs marked by SOX2 (**f**) in xenografts derived from T4121 GSCs expressing shPTPRZ1 or shNT. Data are shown as means ± s.d., **$p$ < 0.01, ANOVA test. Scale bar represents 25 μm.

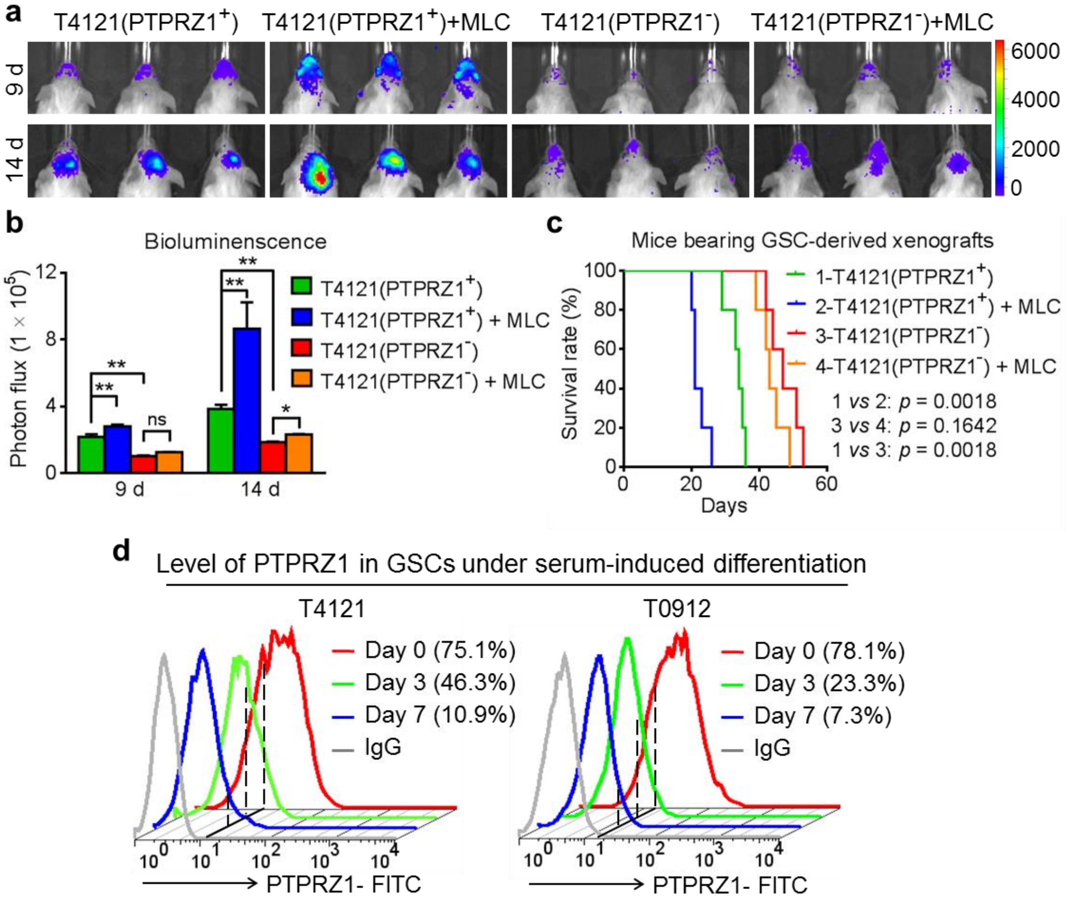


**Supplementary Figure 7.** TAMs promote the tumour growth of PTPRZ1$^+$ glioma cells that are enriched with GSCs.

(**a-b**) Representative bioluminescent images (**a**) and the quantification (**b**) of the intracranial xenografts derived from PTPRZ1$^+$ glioma cells, PTPRZ1$^+$ glioma cells + MLCs, PTPRZ1$^-$ glioma cells or PTPRZ1$^-$ glioma cells + MLCs. Data are shown as means ± s.e.m., **$p$ < 0.01, *$p$ < 0.05, ns, not significant, ANOVA test.

(**c**) Kaplan-Meier analyses of mice bearing PTPRZ1$^+$ glioma cells, PTPRZ1$^+$ glioma cells + MLCs, PTPRZ1$^-$ glioma cells or PTPRZ1$^-$ glioma cells + MLCs. Log-rank test.

(**d**) Flow cytometry analysis of PTPRZ1 expression in GSCs (T4121 and T0912) cultured in serum-induced differentiation medium over a 7-day period.

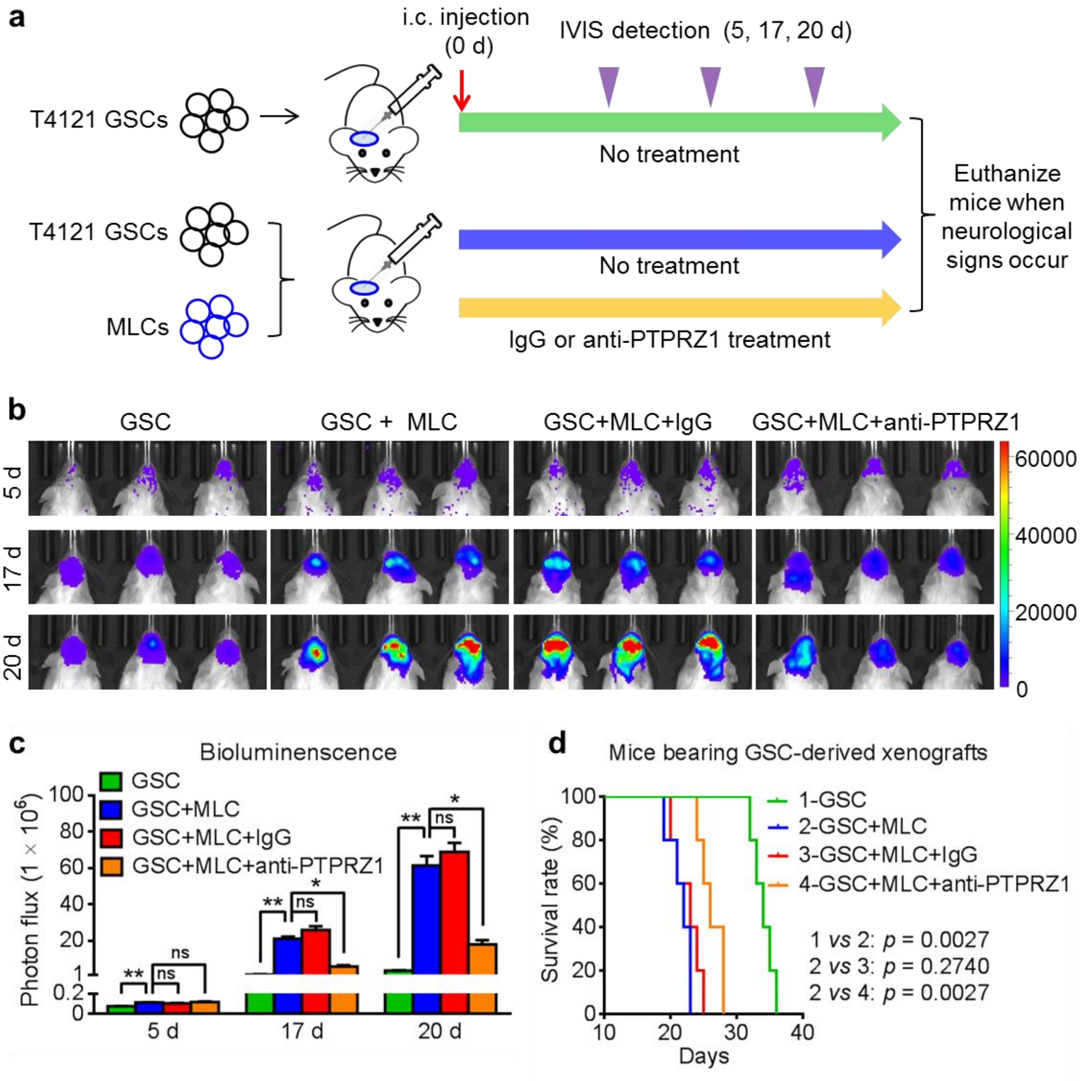


**Supplementary Figure 8.** Anti-PTPRZ1 treatment inhibits GSC tumour growth and prolongs animal survival.

(**a**) Schematic diagram of GSC-driven xenografts co-implanted with MLCs in combination with the treatment of anti-PTPRZ1 antibody. After implantation, mice were left untreated (GSC control or GSC+MLC control) or treated with the anti-PTPRZ1 antibody or isotype IgG every two days until moribund. Tumour growth was monitored through the IVIS bioluminescent imaging system and mice were euthanized when neurological signs occurred.

(**b, c**) Representative bioluminescent images (**b**) and the quantification (**c**) of intracranial GBM xenografts derived from GSC or GSC+MLC treated with anti-PTPRZ1 antibody or isotype IgG control at the indicated time points after cell transplantation. GSCs were transduced with luciferase-expressing vector before implantation. n = 5 per group. Data are shown as means ± s.e.m., *$p < 0.05$, **$p < 0.01$, ns, not significant, ANOVA test.

(**d**) Kaplan-Meier survival analysis of mice implanted with GSC or GSC+MLC in

combination with the treatment of anti-PTPRZ1 antibody. n = 5, log-rank test.

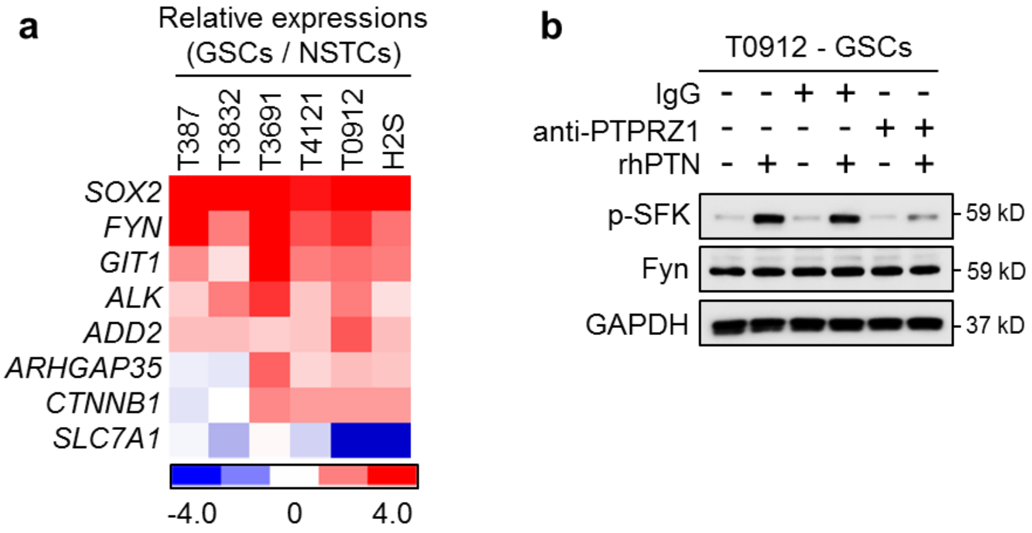


**Supplementary Figure 9.** The PTN-PTPRZ1 signaling regulates Fyn kinase phosphorylation.

(**a**) qRT-PCR analyses of the expressions of seven identified downstream targets of PTPRZ1 in 6 pairs of GSCs and matched NSTCs isolated from primary GBMs. The GSC marker *SOX2* was employed as the positive control. The data were presented as heatmap using Cluster/Java Treeview.

(**b**) Immunoblot analyses of phospho-Src family kinase (p-SFK, Tyr416) and Fyn in T0912 GSCs, showing that rhPTN stimulation markedly increased activating phosphorylation of SFK (p-Tyr416), while the anti-PTPRZ1 antibody treatment largely abrogated SFK activation in GSCs.

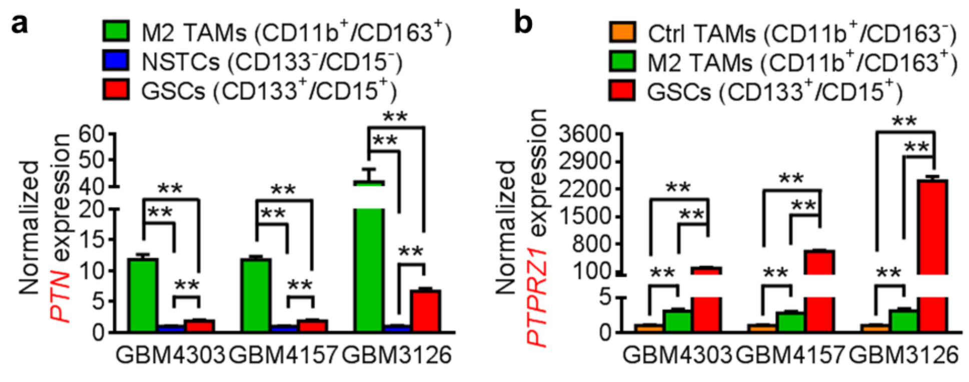


**Supplementary Figure 10.** PTN is preferentially expressed in CD163$^{+}$ M2 TAMs and PTPRZ1 is preferentially expressed in GSCs in human GBMs.

(**a**) qRT-PCR analyses of *PTN* expression in M2 TAMs (CD11b$^{+}$/CD163$^{+}$), NSTCs (CD133$^{-}$/CD15$^{-}$) and GSCs (CD133$^{+}$/CD15$^{+}$) from 3 cases of human GBMs. Data are shown as means ± s.e.m., ***p* < 0.01, ANOVA test.

(**b**) qRT-PCR analyses of *PTPRZ1* expression in M2 TAMs (CD11b$^{+}$/CD163$^{+}$),

control TAMs (CD11b$^{+}$/CD163$^{-}$) and GSCs (CD133$^{+}$/CD15$^{+}$) from 3 cases of human GBMs. Data are shown as means ± s.e.m., ***p* < 0.01, ANOVA test.

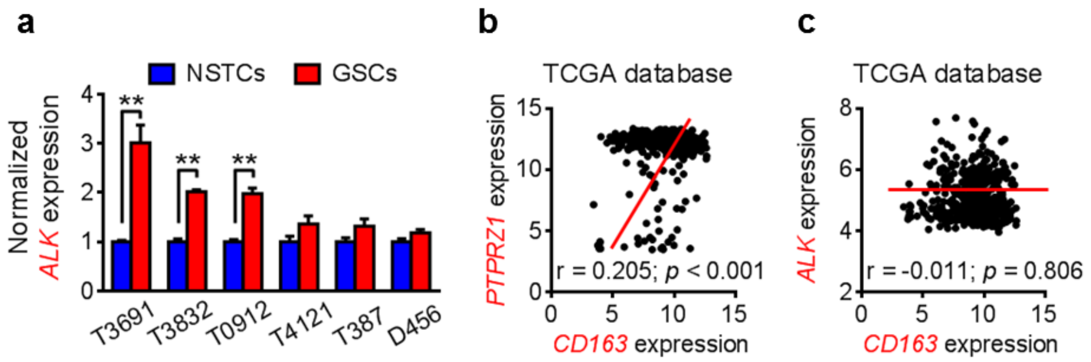


**Supplementary Figure 11.** PTPRZ1 rather than ALK is more likely to be associated with GSC phenotypes and mediates the crosstalk between GSCs and TAMs.

(**a**) qRT-PCR analysis of *ALK* in GSCs and matched NSTCs derived from human GBMs. Data are shown as means ± s.e.m., ***p* < 0.01, student's *t*-test.

(**b-c**) Bivariate correlation analyses of *CD163* and *PTPRZ1* (**b**) or *ALK* (**c**) in human GBMs from the TCGA database. $p < 0.001$ (**b**), $p = 0.806$ (**c**), Pearson *r* test.

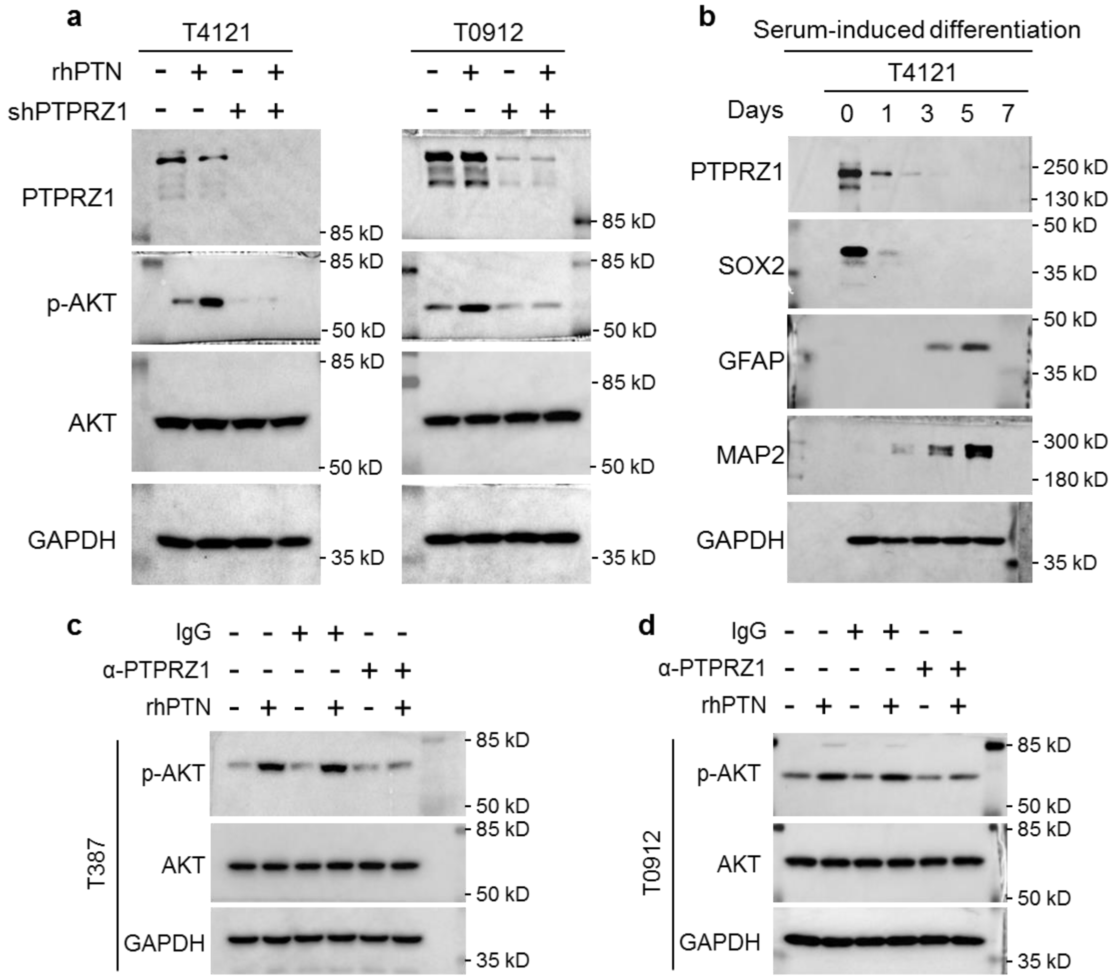


**Supplementary Figure 12.** Original scans of representative full-length blots in the main figures.

(**a**) Immunoblot analyses of PTPRZ1, phospho-AKT (p-Ser473) and total AKT in GSCs expressing shPTPRZ1 or shNT in combination with rhPTN stimulation.

(**b**) Immunoblot analyses of PTPRZ1, the GSC marker SOX2, the astrocytic differentiation marker GFAP and the neuronal differentiation marker MAP2 in GSCs cultured in serum-induced differentiation medium over a 7-day period.

(**c-d**) Immunoblot analyses of phospho-AKT (p-Ser473) and total AKT in T387 GSCs (**c**) and T0912 GSCs (**d**) upon rhPTN and/or anti-PTPRZ1 antibody treatment.

**Supplementary Table 1 Quantifications of SOX2, PTN or CD163 positive cells of in human GBMs.**

| Patient ID | % of $SOX2^{+}$ cells | | % of $CD163^{+}$ cells | | % of $PTN^{+}$ cells | |
|---|---|---|---|---|---|---|
| | Average | Standard Deviation | Average | Standard Deviation | Average | Standard Deviation |
| GBM0358 | 19.20% | 7.33% | 11.40% | 3.97% | 15.60% | 5.03% |
| GBM0867 | 42.80% | 11.01% | 10.20% | 3.83% | 21.00% | 7.00% |
| GBM1305 | 33.20% | 12.03% | 10.00% | 2.92% | 22.00% | 5.66% |
| GBM1435 | 56.60% | 13.65% | 22.20% | 6.30% | 41.20% | 11.10% |
| GBM1866 | 44.40% | 15.53% | 9.20% | 3.70% | 13.20% | 3.56% |
| GBM4668 | 59.80% | 12.91% | 11.20% | 2.77% | 27.00% | 7.00% |
| GBM4981 | 68.00% | 16.08% | 27.60% | 5.94% | 38.60% | 8.20% |
| GBM5450 | 27.80% | 9.86% | 10.40% | 3.44% | 15.20% | 5.07% |
| GBM2399 | 67.40% | 14.84% | 24.20% | 7.36% | 26.20% | 6.65% |
| GBM6702 | 45.80% | 8.17% | 9.20% | 2.49% | 17.80% | 4.82% |
| GBM7010 | 59.80% | 14.84% | 11.40% | 5.03% | 19.40% | 5.03% |
| GBM7511 | 34.40% | 13.13% | 16.00% | 6.32% | 16.00% | 3.87% |
| GBM7857 | 68.00% | 14.02% | 19.60% | 6.58% | 25.80% | 6.98% |
| GBM8847 | 62.20% | 12.87% | 34.40% | 11.59% | 33.20% | 8.73% |
| GBM9146 | 30.40% | 10.48% | 10.00% | 3.54% | 15.40% | 4.67% |
| GBM0156 | 73.40% | 15.44% | 22.60% | 6.43% | 31.20% | 8.67% |
| GBM4730 | 12.80% | 4.97% | 3.60% | 1.95% | 4.20% | 2.59% |
| GBM3126 | 58.80% | 14.65% | 11.20% | 4.09% | 14.20% | 5.07% |
| GBM6079 | 15.20% | 6.18% | 1.00% | 0.71% | 2.60% | 2.07% |
| GBM7503 | 69.80% | 13.41% | 24.80% | 6.98% | 32.80% | 7.26% |

Abbreviations: GBM, Glioblastoma.

**Supplementary Table 2** The overlapped upregulated genes in GSCs relative to NSTCs from both GDS3885 and GSE54791 gene expression profiles.

| Gene | GSCs/NSTCs (GDS3885) | GSCs/NSTCs (MGG8) | GSCs/NSTCs (MGG6) | GSCs/NSTCs (MGG4) |
|---|---|---|---|---|
| ***PTPRZ1*** | **284.2** | **106.6139** | **624.8375** | **132.7656** |
| *FABP7* | 147.74 | 10.08799 | 34.77892 | 194.9979 |
| *GPM6B* | 118.175 | 30.8512 | 81.20673 | 62.398 |
| *FXYD6* | 83.62 | 25.1358 | 15.1378 | 21.46844 |
| *S100B* | 77.78 | 170.6224 | 265.7078 | 205.7967 |
| *OLIG1* | 58.98 | 357.0636 | 282.235 | 115.3386 |
| *PMP2* | 39.25 | 159.9883 | 251.1959 | 49.80343 |
| *SOX8* | 34.7 | 42.76171 | 139.8296 | 112.4571 |
| *MLC1* | 33.49 | 108.3228 | 20.27441 | 10.52407 |
| *OLIG2* | 30.22 | 162.8567 | 254.4103 | 143.4052 |
| *MAP2* | 29.795 | 35.55822 | 97.47352 | 17.4938 |
| *SOX2* | 29.32333 | 51.12857 | 44.66943 | 37.82426 |
| *NCAN* | 25.98 | 83.68638 | 283.8979 | 579.0954 |
| *LHFPL3* | 24.34 | 67.79991 | 27.8575 | 3.173257 |
| *INSM1* | 21.76 | 12.17771 | 283.7458 | 3.984551 |
| *AIF1L* | 18.94 | 44.08958 | 109.0063 | 25.56094 |
| *RNF157* | 18.51 | 81.00799 | 23.52447 | 16.44765 |
| *ABAT* | 18.085 | 9.232167 | 13.80151 | 3.371962 |
| *SLAIN1* | 17.83 | 13.69892 | 7.09865 | 75.33327 |
| *C1orf61* | 17.82667 | 126.8359 | 283.226 | 15.18785 |
| *SCRG1* | 17.61 | 109.0686 | 135.7286 | 14.57756 |
| *LOC100506421* | 17.27 | 19.24312 | 105.978 | 12.66922 |
| *GNG7* | 17.07 | 12.47806 | 14.7929 | 15.6755 |
| *SLC35F1* | 16.47 | 42.01335 | 38.04914 | 25.47074 |
| *PTN* | 16.3 | 3.218268 | 4.174727 | 17.20702 |
| *TTYH1* | 13.98 | 109.5139 | 232.4419 | 83.70676 |
| *HES6* | 13.87 | 8.323347 | 12.75646 | 28.96647 |
| *GPRC5B* | 13.32 | 10.61411 | 8.487462 | 46.80197 |
| *KIF5C* | 11.86 | 4.294404 | 4.780516 | 8.037809 |
| *LPHN3* | 11.4175 | 82.02413 | 7.043419 | 36.52589 |
| *ASCL1* | 11.35333 | 268.0327 | 147.1108 | 135.3764 |
| *CPVL* | 11.27 | 40.15137 | 8.964373 | 19.64955 |
| *CHD7* | 10.905 | 6.792906 | 16.52242 | 42.43733 |
| *CKB* | 10.82 | 18.0026 | 5.516465 | 3.483312 |
| *COL9A3* | 10.66 | 113.2781 | 189.9681 | 37.42095 |
| *PEG3* | 9.555 | 9.558056 | 47.7629 | 5.20987 |
| *GAS7* | 9.462 | 34.11968 | 137.8827 | 61.28899 |
| *BMP7* | 9.405 | 125.0853 | 40.72697 | 182.4509 |
| *CERS1* | 9.07 | 19.80103 | 21.08558 | 9.636814 |
| *PREX1* | 8.71 | 6.960822 | 11.89368 | 5.536499 |
| *LSAMP* | 8.605 | 21.52411 | 4.926939 | 14.56914 |
| *KCNQ2* | 8.13 | 302.386 | 20.62636 | 44.32335 |
| *NPAS3* | 8.07 | 6.296905 | 5.1753 | 3.883754 |
| *MAP3K1* | 7.6 | 7.754265 | 12.98774 | 4.105784 |

| | | | | |
|---|---|---|---|---|
| *TUBB2B* | 6.745 | 9.161784 | 10.72267 | 15.99255 |
| *C8orf46* | 6.695 | 39.45976 | 32.31601 | 6.751572 |
| *PPP1R14C* | 6.37 | 14.07802 | 5.721182 | 229.8719 |
| *TMEM198* | 6.35 | 19.33576 | 3.399607 | 5.234361 |
| *ELOVL2* | 6.1 | 42.85471 | 27.38499 | 4.831071 |
| *ETV4* | 6.07 | 4.958348 | 13.6188 | 3.966295 |
| *ATP1B2* | 6.05 | 21.21876 | 209.9979 | 37.80505 |
| *NLGN4X* | 5.98 | 43.24325 | 3.317387 | 24.92908 |
| *TNFRSF19* | 5.79 | 50.53871 | 5.157408 | 6.115593 |
| *B3GAT1* | 5.62 | 5.356394 | 49.04268 | 94.54959 |
| *FOS* | 5.51 | 236.4087 | 8.959965 | 7.295587 |
| *NOTCH1* | 5.33 | 10.20499 | 7.901075 | 4.301373 |
| *FGFBP3* | 5.08 | 73.51515 | 19.95837 | 26.4541 |
| *KIF21A* | 5.055 | 5.89221 | 19.3974 | 10.84724 |
| *CYFIP2* | 4.93 | 4.4851 | 3.384014 | 4.87878 |
| *MANEAL* | 4.64 | 14.36268 | 3.370203 | 4.645122 |
| *GNG4* | 4.573333 | 30.19385 | 24.72519 | 12.94752 |
| *LDLRAD3* | 4.5 | 3.4707 | 7.860397 | 12.45414 |
| *CPXM1* | 4.44 | 35.40068 | 430.711 | 50.68503 |
| *RTKN* | 4.42 | 3.368384 | 3.651618 | 3.41792 |
| *METRN* | 4.04 | 17.44902 | 16.00526 | 4.948844 |
| *SBK1* | 3.95 | 7.468824 | 9.872813 | 47.32056 |
| *GOLM1* | 3.9 | 4.229261 | 4.823762 | 4.579978 |
| *DHRS13* | 3.82 | 18.06053 | 10.81561 | 3.301548 |
| *FAM19A5* | 3.72 | 35.7709 | 6.085051 | 132.8813 |
| *CA14* | 3.69 | 220.3728 | 4.102199 | 12.71834 |
| *ISYNA1* | 3.61 | 15.65274 | 7.843448 | 3.854048 |
| *EPHB3* | 3.59 | 39.80339 | 5.250148 | 6.669024 |
| *EYA2* | 3.51 | 8.578824 | 449.6803 | 135.5749 |
| *TRAF4* | 3.49 | 6.376761 | 3.737991 | 3.578133 |
| *DBI* | 3.46 | 5.918128 | 8.199854 | 3.230083 |
| *BMP8B* | 3.44 | 77.72659 | 31.13967 | 23.74176 |
| *NDRG2* | 3.33 | 37.44697 | 41.96949 | 84.37755 |
| *LPPR5* | 3.25 | 74.98744 | 126.3963 | 36.87843 |
| *TMEM121* | 3.24 | 4.180507 | 4.795929 | 4.653788 |
| *SCG3* | 3.09 | 49.7505 | 33.70296 | 157.5403 |
| *NAT8L* | 3.07 | 57.58645 | 52.47291 | 116.3892 |
| *KCNF1* | 3.05 | 7.20778 | 6.873389 | 18.44829 |
| *CTXN1* | 3.02 | 3.959728 | 4.972054 | 5.23158 |

Abbreviations: GSCs, glioblastoma stem cells; NSTCs, non-stem tumor cells. MGG4, 6 and 8, primary GBM cell lines from dataset GSE54791.

**Supplementary Table 3** Pathological characteristics of human GBMs used in this study.

| Specimen | Histopathology | WHO Grade | Gender | Age | Predominant side of tumor location | Predominant lobe of tumor location | Extent of surgical resection |
|---|---|---|---|---|---|---|---|
| GBM4730 | GBM | IV | Male | 77 | Left | Frontal | GTR |
| GBM5385 | GBM | IV | Male | 64 | Right | Occipital | GTR |
| GBM4303 | GBM | IV | Male | 61 | Left | Frontal | GTR |
| GBM4157 | GBM | IV | Male | 50 | Left | Frontal | GTR |
| GBM3126 | GBM | IV | Male | 62 | Right | Parietal | GTR |
| GBM2833 | GBM | IV | Female | 25 | Left | Frontal | GTR |
| GBM2399 | GBM | IV | Male | 44 | Right | Temporal | PR |
| GBM7503 | GBM | IV | Male | 67 | Left | Parietal | GTR |
| GBM4668 | GBM | IV | Female | 51 | Right | Parietal | GTR |
| GBM0358 | GBM | IV | Male | 53 | Both | Frontal | PR |
| GBM0867 | GBM | IV | Male | 61 | Right | Frontal | PR |
| GBM1218 | GBM | IV | Male | 50 | Left | Temporal | PR |
| GBM1305 | GBM | IV | Male | 40 | Right | Temporal | GTR |
| GBM1435 | GBM | IV | Male | 58 | Right | Temporal | GTR |
| GBM1866 | GBM | IV | Male | 64 | Right | Temporal | PR |
| GBM4798 | GBM | IV | Female | 33 | Left | Temporal | GTR |
| GBM4981 | GBM | IV | Female | 64 | Right | Parietal | GTR |
| GBM5450 | GBM | IV | Male | 61 | Left | Frontal | GTR |
| GBM6702 | GBM | IV | Male | 71 | Right | Temporal | GTR |
| GBM7010 | GBM | IV | Male | 58 | Right | Temporal | PR |
| GBM7511 | GBM | IV | Female | 58 | Left | Temporal | PR |
| GBM7857 | GBM | IV | Male | 51 | Right | Frontal | GTR |
| GBM8847 | GBM | IV | Male | 47 | Right | Frontal | GTR |
| GBM9146 | GBM | IV | Female | 25 | Left | Occipital | PR |
| GBM9387 | GBM | IV | Male | 53 | Right | Parietal | GTR |
| GBM0156 | GBM | IV | Male | 77 | Right | Temporal | GTR |
| GBM6079 | GBM | IV | Male | 48 | Left | Frontal | GTR |
| GBM3446 | GBM | IV | Male | 75 | Left | Temporal | PR |
| GBM9360 | GBM | IV | Female | 64 | Left | Parietal | GTR |

Abbreviations: GBM, Glioblastoma; GTR, Gross total resection; PR, Partial resection.

**Supplementary Table 4** Short hairpin RNA sequences used for lentiviral vector construction.

| Vector | Sequence (5' to 3') |
|---|---|
| shNT (for PTN) | CCGGCAACAAGATGAAGAGCACCAACTCGAGTTGGTGCTCTTCATCTTGTTGTTTTT |
| shPTN-1 | CCGGTCAGCAAACAGGATCAGTTAACTCGAGTTAACTGATCCTGTTTGCTGATTTTTG |
| shPTN-2 | CCGGAGGCAAGAAACAGGAGAAGATCTCGAGATCTTCTCCTGTTTCTTGCCTTTTTT |
| shNT (for PTPRZ1) | TTCTCCGAACGTGTCACGT |
| shPTPRZ1-1 | GCACAAGAATCGATACATA |
| shPTPRZ1-2 | CGAAGGAACTGTCAACATA |

**Supplementary Table 5** Primers used for qRT-PCR analyses in this study.

| Gene | Forward primer (5' to 3') | Reverse primer (5' to 3') |
|---|---|---|
| *CD163* | TTTGTCAACTTGAGTCCCTTCAC | TCCCGCTACACTTGTTTTCAC |
| *PTN* | GGAGCTGAGTGCAAGCAAAC | CTCGCTTCAGACTTCCAGTTC |
| *ADM* | ATGAAGCTGGTTTCCGTCG | GACATCCGCAGTTCCCTCTT |
| *CDL14A1* | TTCAGACTGGTTCGGCATTTC | CTGTGCAAGACCAATTCGTGT |
| *GDF3* | TCCTGGAGATACTGGTCAAAGAA | GAGCATCTTAGTCTGGCACAG |
| *IGFBP3* | AGAGCACAGATACCCAGAACT | GGTGATTCAGTGTGTCTTCCATT |
| *SPARCL1* | CCAACTGAAGGTACATTGGACAT | CTGTGAAGGAACTAACACCAGG |
| *DNER* | AAGGCTATGAAGGTCCCAACT | CTGAGAGCGAGGCAGGATTT |
| *IGFBP2* | GACAATGGCGATGACCACTCA | CAGCTCCTTCATACCCGACTT |
| *MMP10* | TCAGTCTCTCTACGGACCTCC | CAGTGGGATCTTCGCCAAAAATA |
| *TFPI2* | TCCTGCCCCTAGACTACGG | CTCCCAGGTGTAGAAATTGTTGG |
| *Fizz1* | AGAGTACAGTCCCTCTCC | AACCACAGCCATAGCCACAA |
| *Arg1* | TGGACAGACTAGGAATTGGCA | CCAGTCCGTCAACATCAAAACT |
| *CD206* | CGATCCGACCCTTCCTTGAC | TGTCTCCGCTTCATGCCATT |
| *iNOS* | TTCAGTATCACAACCTCAGCAAG | TGGACCTGCAAGTTAAAATCCC |
| *MHCII* | GAGCAGGTTAAACATGAGTGTCA | CTCTCCACAACCCCGTAGT |
| *PTPRZ1* | GCCTGGATTGGGCTAATGGAT | CAGTGCTCCTGTATAGGACCA |
| *SOX2* | TACAGCATGTCCTACTCGCAG | GAGGAAGAGGTAACCACAGGG |
| *ADD2* | ACACCTATGTCACGTTGAGAGT | TCTCCCAGAATGTTCACCTTGA |
| *FYN* | TGGAGGTGTGAACTCTTCGTC | TCTGTCCGTGCTTCATAGTCA |
| *CTNNB1* | AGCTTCCAGACACGCTATCAT | CGGTACAACGAGCTGTTTCTAC |
| *GIT1* | AGCCTTGACTTATCCGAATTGG | CACCTCGTCATACACGTCCA |
| *SLC7A1* | GCCTGTGCTATGGCGAGTTT | ACGCTTGAAGTACCGATGATGTA |
| *ALK* | TCTCATCGCAGCCGATATGG | GGCATCTCCTTAGAACGCTCT |
| *ARHGAP35* | GACTTTTCAACCTCATCGAAGCA | GCTTTCCGTCTGGCATTTGTT |
| *ACTB* | CTCCTCCGAGTCAACAGATTCA | CAACAGCTTCTGAGGTAGGGA |
| *GAPDH* | AAGGTGAAGGTCGGAGTCAAC | GGGGTCATTGATGGCAACAATA |
